# Review of Progress in Shape Memory Epoxies and Their Composites

**DOI:** 10.3390/polym10010034

**Published:** 2017-12-29

**Authors:** József Karger-Kocsis, Sándor Kéki

**Affiliations:** 1 Department of Polymer Engineering, Faculty of Mechanical Engineering, Budapest University of Technology and Economics, Műegyetem rkp. 3, H-1111 Budapest, Hungary; 2 MTA–BME Research Group for Composite Science and Technology, Műegyetem rkp. 3, H-1111 Budapest, Hungary; 3 Department of Applied Chemistry, University of Debrecen, Egyetem tér 1, H-4032 Debrecen, Hungary; keki.sandor@science.unideb.hu

**Keywords:** shape memory epoxy resin, multishape memory, two-way shape memory, shape memory assisted self-healing, (nano)composite, hybrid composite, modelling, vitrimer chemistry, shape memory alloy, network structure, multifunctionality, 4D printing

## Abstract

Shape memory polymer (SMP) is capable of memorizing one or more temporary shapes and recovering successively to the permanent shape upon various external stimuli. Beside of the above mentioned one-way variants, also two-way shape memory polymers (SMPs) and shape memory (SM) systems exist which feature a reversible shape change on the basis of “on-off switching” of the external stimulus. The preparation, properties and modelling of shape memory epoxy resins (SMEP), SMEP foams and composites have been surveyed in this exhaustive review article. The underlying mechanisms and characteristics of SM were introduced. Emphasis was put to show new strategies on how to tailor the network architecture and morphology of EPs to improve their SM performance. To produce SMEPs novel preparation techniques, such as electrospinning, ink printing, solid-state foaming, were tried. The potential of SMEPs and related systems as multifunctional materials has been underlined. Added functionality may include, among others, self-healing, sensing, actuation, porosity control, recycling. Recent developments in the modelling of SMEPs were also highlighted. Based on the recent developments some open topics were deduced which are merit of investigations in future works.

## 1. Introduction

Shape memory (SM) polymers (SMPs) and composites thereof are emerging smart materials in different applications, especially in biomedical, construction engineering and aerospace fields [1,2,3,4,5]. SMPs may adopt one (dual-shape), two (triple-shape) or several (multi-shape) stable temporary shapes and recover their original (permanent) ones in dual-shape or the previous temporary ones in multi-shape versions upon the action of an external stimulus. The external stimulus may be temperature (thermo-responsive; temperature variation triggered by direct or indirect ways), chemical environment (water, pH, solvent, redox conditions, etc.), irradiation (light at different wavelength) and mechanical load (mechano-responsive) [6]. In most cases, however, the SMPs are thermo-sensitive (thermally activated) ones. The “switching” or transformation temperature (*T*_trans_), enabling the material to return from the temporary to its permanent shape, is linked with the glass transition (*T*_g_) or the melting temperature (*T*_m_) [4,7]. Therefore, the SMPs are often subdivided based on their switch types into *T*_g_- or *T*_m_-based SMPs. As “switches,” however, other reversible mechanisms such as liquid crystallization/melting, supermolecular assembly/disassembly, irradiation-induced reversible network formation, formation and formation/disruption of a percolation-type network, may serve [4,5,7]. The permanent shape is guaranteed by physical (entanglement, interpenetrating networks) or chemical networks (composed of permanent or temporary covalent bonds) which are hold together by “net points.” The temporary shape is set by mechanical deformation above *T*_trans_ and it is fixed by cooling below *T*_trans_ whereby maintaining the mechanical load. However, the deformation temperature may be below *T*_g_ or *T*_m_ of the corresponding polymer, as well.

For thermosets, such as epoxies (EP), the transformation temperature is the *T*_g_. During setting the temporary shape, the segments between the crosslinks adapt to the external load via conformational rearrangements. The strain energy, stored by this way, is released when the material is unloaded and heated above its *T*_g_ *via* which the permanent shape is restored. Thus, the shape change is of entropic nature. It was demonstrated by nanoindentation that SM can persist in very small, nanometre-size volumes even in crosslinked EP networks [8].

Discovery of the vitrimer chemistry by the group of Leibler [9] reshaped the landscape in the field of SM thermosets. Vitrimers represent a new class of crosslinked polymers which possess the interchange of covalent bonds above a temperature *T*_p_ (subscript p refers to plasticity) located above the *T*_g_. Polymer plasticity, allowing the reshaping of polymers permanently, is triggered by covalent bond exchange resulting in a load-induced topographical rearrangement of the network. So, the shape change is not recoverable and not associated with a change in the entropy. By contrast to the former introduced “elasticity”-based SM behaviour (including shape erasing and (re)programming possibilities), the “plasticity”-based SM refers to permanent and cumulative reshaping possibility. Combination of the two concepts opens a new horizon for the development of SMEPs [10].

All what is disclosed above is related to one-way (1W) SMPs. This means that the external stimulus activates only the change from the temporary to the permanent shape (dual-shape) or from one temporary to the other one (multi-shape version). Apart from 1W, also two-way (2W) SMP systems, featuring a reversible shape change on the basis of “on-off” switching of the external stimulus, exist, as introduced later.

**Figure 1:**
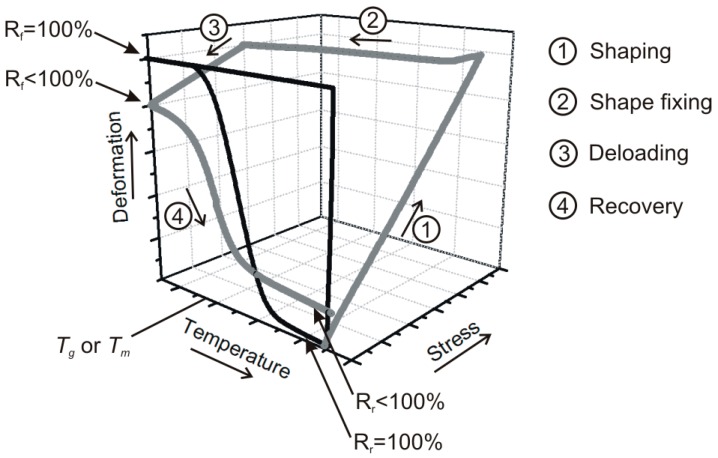
Single SM cycles of a 1W-SM polymer (black) and its composite (grey), schematically.

The shape memory (SM) properties are typically quantified by the shape fixity (*R*_f_) and shape recovery ratios (*R*_r_). *R*_f_ means the extent of fixing of the externally applied deformation in the temporary shape. Its value is 100% when the applied deformation, introduced above *T*_trans_, is fully kept below *T*_trans_ in the temporary shape. The deformation modes cover tension, compression, bending and torsion. *R*_r_ is the percentage of the recovery of the original shape when the material is heated in stress-free state (unconstrained) above *T*_trans_ subsequently. *R*_r_ = 100% when the original shape of the material is fully restored. SM properties are often determined in cyclic (one or more) thermomechanical tests performed under stress- or strain-controlled conditions. Keeping the temporary shape during reheating enables the material to behave as a force actuator. Determination of the force, stress occurs under constrained conditions, i.e., when the specimen’s shape is fixed [11,12]. Figure 1 displays the course of SM thermomechanical tests under unconstrained conditions. Figure 1 also highlights the major benefit of SM composites: strongly enhanced recovery stress (when measured under constraint) generally at cost of shape fixing and recovery (determined in stress-free, unconstrained state). Beside *R*_f_ and *R*_r_, further SM characteristics, such as the temperature interval of recovery, recovery rate and recovery force, can be measured. It should be underlined here that the *R*_f_ and *R*_r_ values reported in this article were measured under different conditions and thus their values cannot be directly compared.

A large body of works was already dealing with various aspects of SMPs and related composites [4,5,7,13,14,15,16]. Moreover, topic-relevant excellent reviews on shape memory thermosets [17] and even epoxies (SMEPs) [18,19] are available. The actuality of this review is due to recent advancements in the field of SMEPs and their composites. Recent works follow novel strategies for network modification, shape change triggering, composites’ structuring and preparation, creation of multifunctional polymers. The related aspects are covered by this review. For the sake of completeness, however, relevant former works are also included in this survey. During presentation of the results emphasis was put on the actual structure of the neat SMEPs and their various composites. On the other hand, less information was given on the application potential of SMEP systems because this issue is already well covered in the literature. In this respect, the interested reader is advised to have a look at the titles of the cited works.

## 2. One-Way, Dual-Shape Memory Epoxy (EP) Formulations

Preferred thermoset SMPs are EP-based ones. EPs are selected owing to their favourable properties (heat and chemical resistance, high stiffness, good adhesion to various substrates) and versatility (easy tuning of *T*_g_ and of the stiffness in the glassy and rubbery states). Next, we shall introduce the basic strategies on how to tailor the network structure and thus the performance of SMEPs in order to widen their potential applications.

### 2.1. (Co)network Structure

As mentioned before, the chemistry of EP curing is versatile [20] enough to tailor the *T*_g_ and thus also the *T*_trans_, upon request. One of the basic formulation tools is the EP/hardener ratio: whether it is stoichiometric or not. Off-stoichiometry itself results in reduced *T*_g_ values [21]. It is practiced usually by adding less hardener than required by the stoichiometric ratio rather to adding more. For example, *T*_g_ values between 45 and 145 °C can be measured for a bisphenol A-based bifunctional EP cured with aromatic diamine when the curing degree is varied between 50 and 100% (corresponding to the stoichiometry) [22]. This change is accompanied with an almost threefold increase in the crosslinking density. Researchers, however, prefer to use fully cured EPs, formulated by stoichiometric amount of hardener. Even in the latter case, there are numerous possibilities to manipulate the build-up of the network along with the associated viscoelastic properties. Network architecturing traditionally achieved by suitable alterations in the hardeners, EP resins or in both.

**Figure 2:**
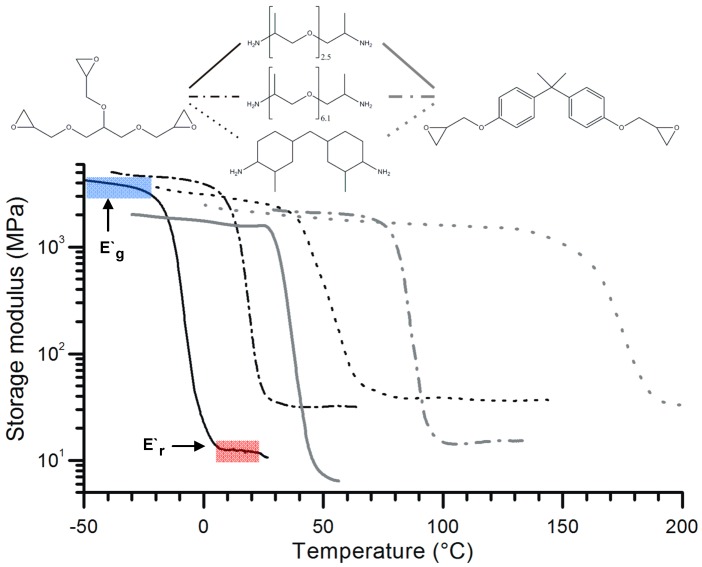
Effects of selected formulations (types of EP resins and hardeners) on the viscoelastic response of amine-cured EPs.

For amine-cured EPs several possibilities exist, for example, combination of mono- and dysfunctional amines [19,23], the same type of diamine but with various chain length [19,23], common use of aliphatic and aromatic EPs [19,24], different aromatic amines also at off-stoichiometry [25] and their variations. The combined use of mono- and difunctional amines thereby the chain length of the monoamine is long enough for entangling, may result in a network composed of chemical and physical (entanglements) crosslinks [26]. To improve the thermal stability and mechanical properties of SMEPs Sun et al. [27] have developed a two-stage curing method using multifunctional amine in the first (at room-temperature) and a boron-containing latent curing agent in the second (at elevated temperature) step. For the SM function the first curing was responsible whereas the second curing step was just to enhance the properties. Figure 2 gives an impression on the temperature range in which the viscoelastic properties (*T*_g_, glassy and rubbery storage moduli: *E*_g_′ and *E*_r_′, respectively) of amine-cured EPs may be tailored.

Similar versatility exists also for anhydride cured EPs [20] albeit anhydrides were less frequently used compared to amine for EP curing [28,29,30,31]. The possible reason behind this fact is that the *E*_g_/*E*_r_ ratio is much lower for anhydride-cured than for amine-cured version. It is usually accepted that this ratio should be in the range of several hundred for a polymer to exhibit good SM properties. To overcome this problem long chain anhydrides [28,29] or aliphatic epoxies [30] are incorporated into the network to reduce its rigidity. A further tool of network flexibility is given by changing the stoichiometric ratio. 

A new impetus to anhydride-cured SMEPs yielded the vitrimer chemistry [9,31] which introduces plasticity in the crosslinked networks via exchangeable chemical bonds. Such networks are also termed as covalent adaptable networks [32], which may be further grouped into dissociative and associative variants [31]. The associative version was demonstrated for anhydride- and acid-cured epoxy systems (catalytic carboxylate transesterification reaction) in which also the term “vitrimer” was coined [9]. *T*_p_, linked with the course of the exchange reactions, may be either above or below the *T*_g_, however, for SM function only the former is relevant. Since the possibility of carboxylate transesterification is a specific feature of anhydride/acid-cured EPs, it has been explored with respect to SM properties, as well. Here the permanent shape is generated at or above *T*_p_ and fixed by cooling below *T*_p_. The temperature range for temporary shapes is between *T*_g_ and *T*_p_. Altuna et al. [33] cured bisphenol A-based EP (DGEBA) with different tri- and dicarboxylic acids resulting in *T*_g_s in the range of 51–62 °C. Good (>99%) *R*_f_ and *R*_r_ values were measured for these vitrimers in torsional tests. However, not only the carboxylate transactions can be exploited in SMEPs. The dynamicity of the disulphide linkage may be exploited in SMEP vitrimers as shown by Ma et al. [34]. The cited authors introduced this linkage by the amine hardener 4,4′-disulfanediyl dianiline in an isosorbide-derived EP resin.

The dissociative covalent adaptive network is best represented when it contains thermally reversible Diels-Alder (DA) groups. Pioneering work in this field was done already in 2002 [35]. Note that DA adducts from furan and maleimide groups form usually below 90 °C and their decomposition (retro DA reaction) take place at above 110 °C. Albeit thermal mending and reprocessability are the common targets with DA adducts in the network, the related epoxy-based polymers exhibit good SM behaviour [36]. The dynamic equilibrium of the DA reaction was found as further tool to trigger topological rearrangements within the network of crosslinked systems containing EP constituents [37]. Zhang et al. [37] concluded that the dynamicity of the DA reaction can be exploited for complex shape manipulation thereby making use of activation of the chain segments (elasticity) and DA reversible bonds (plasticity) [10]. Possibilities to exploit the inserting of reversible binding groups in SMPs were recently summarized by Lewis and Dell [38].

It is noteworthy that beside the traditional amine- and anhydride-type curing, also others, such as thiol-epoxy [39], were explored with respect to SM behaviour.

A further tool to tune the network of SMEPs is given by the molecular structure of the EP resins themselves. In the literature many examples can be found for the combinations of hydroepoxy (cycloaliphatic type) and aliphatic EP [40,41,42], aromatic diepoxide and aromatic monoepoxide [21], aromatic and aliphatic EPs [43], similar epoxies with different molecular weights [44], synthesis of EPs containing rigid and flexible units [45], and the like. High-temperature resistant novel EPs with and without liquid crystalline characteristics were also synthesized and their SM behaviour checked [46,47,48].

Nowadays, considerable efforts are in progress to improve the SM performance via creations of different co-networks. Co-networks are chemically crosslinked networks in which none of the constituents forms a continuous phase. This definition does not exclude, however, the possible presence of domains which are rich in one or in other of the constituents. For the creations of EP-containing co-networks with SM properties the epoxy-isocyanate (oxazolidone formation) and epoxy-isocyanurate (oxazolidinone formation) reactions were used [49,50]. The oxazolidone chemistry is a useful way to tailor the hard segments in polyurethanes (PUs) [49]. Note that segmented PUs represent the most versatile and mostly researched family of SMPs [51,52]. Nowadays, the R & D works on shape memory polyurethanes are focused on potential medical applications. The related SM polyurethanes, having a *T*_trans_ matched with the body temperature, are often synthesized using bio-based compounds [53,54,55,56].

The co-network formation of hybrid resins containing EP, cyanate ester and phenol-terminated diol is a very complex process owing to the onset of different chemical reactions resulting in an inhomogeneous co-network. This is well manifested in a broad range of the mechanical loss factor (tanδ), which may even show two peaks. The latter, assigned to polyisocyanurate-rich (high temperature) and polyol-rich segments (low temperature), is a clear appearance of incompatibility. *R*_r_ increased with increasing *E*_g_/*E*_r_ ratio for this hybrid by contrast to the recovery time that showed an adverse trend [50]. Ariraman et al. [57] reacted a cyanate ester (in various amounts) with DGEBA thereby creating a co-network containing oxazoline groups in the chain segments and triazine rings as netpoints. Both *R*_f_ and *R*_r_ increased with increasing content of the cyanate ester.

Benzoxazines can well be combined with EPs because polybenzoxazine, formed by ring-opening polymerization, bears phenolic hydroxyl groups which may react with the epoxy groups. In the case of amine curing agents of the benzoxazine/EP blend the oxazine ring is cleaved by the amine [58,59] resulting in a heterogeneous (polybenzoxazine and epoxy rich domains) co-network. Amine-cured SMEP formulations composed of aliphatic and aromatic EPs and bisphenol-A/aniline-based benzoxazine were produced by Rimdusit et al. [60,61] and tested for SM performance. *R*_f_ was close to 100% by contrast to *R*_r_ showing values of 90% or lower. Major benefit of benzoxazine incorporation was a strong increase in the recovery stress. Various click reactions may also be used to create co-networks for SM function. Sunitha et al. [62] synthesized azide functionalized EP and propargylated novolac and coupled them through triazol rings via azide-alkyne-type “click reaction.” This material showed *R*_f_ and *R*_r_ values of 99% and 90%, respectively.

Hyperbranched or star-shape polymers bearing reactive groups with epoxy are often used to toughen the EP [63]. In their presence a co-network, eventually accompanied with strong phase segregation, is formed that markedly enhance the ductility of the parent EP. EPs modified with hyperbranched polyethyleneimine (multifunctional amine-type crosslinking agent) showed excellent shape fixity and shape recovery of 98% along with a fast shape recovery rate [64,65]. Alternatively, EP can be used as minor component to modify hyperbranched polymers, such as polyurethane [66]. The corresponding EP-modified hyperbranched polyurethane exhibited shape recovery values between 90% and 98%.

Rousseau [67] pinpointed that the excellent *R*_f_ and *R*_r_ values (≥95%) for SMEPs are due their high *E*_g_ and beneficial network-given rubber elasticity above *T*_g_. Like all polymers, also SMEPs are viscoelastic materials. This means that the properties depend on both temperature and time. In the time scale especially heating/cooling and physical aging [68] effects should be taken into consideration. Viscoelasticity implies a strong influence of the selection of *T*_trans_ on the SM properties. *T*_trans_ is traditionally higher than *T*_g_ (≥15–20 °C). *T*_trans_ may be, however, at the vicinity or even below that of *T*_g_. It is known that the network deformability goes through a maximum when passing *T*_g_ from the glassy to the rubbery state. Feldkamp and Rousseau [69] showed a fivefold increase in the stress-strain response for an EP when it was deformed at the onset of *T*_g_ instead of above *T*_g_. The thermomechanical recovery process highly depends whether *T*_trans_ was above or below *T*_g_ of the corresponding EP. Liu et al. [70] demonstrated in a detailed study that the stress-strain response of shaping above *T*_g_ agrees well with that of the recovery, triggered always above *T*_g_. By contrast, when shaping was done below *T*_g_, the recovering stress-strain response differed markedly from the one measured at the shaping temperature. In the latter case the recovery stress upon flexure peaked at *T*_g_ with a maximum value much smaller than measured upon shaping at *T*_trans_. Moreover, with increasing cooling rate during shape fixing the temperature needed to reach *R*_f_ = 100% was shifted toward lower temperatures. This was associated with an increasing level of the recovery stress. Understanding the coupling between cooling (shape fixing) and heating rates (shape recovery) and the evolution of the recovery response is a key issue for many applications. This aspect was addressed in a recent in depth study by Pandini et al. [21]. The cited authors studied the effects of network architecture of the SM behaviour of SMEPs under both dynamic and isothermal recovery conditions. Temporary shaping of the resins was done at *T*_trans_ < *T*_g_, termed to as “cold working.” This kind of thermomechanical programming was argued to be less time-consuming, easier to perform and bringing additional benefits (improved stress-strain behaviour) than the traditional one at *T*_trans_ > *T*_g_. It was found that *R*_f_ decreased with increasing difference between *T*_g_ and *T*_trans_ (i.e., *T*_g_ − *T*_trans_). It is noteworthy that the *R*_f_ data for the “cold worked” EPs were somewhat below those of the traditionally deformed (i.e., *T*_trans_ > *T*_g_) counterparts. A further conclusion of Pandini et al. [21] was that the SM recovery behaviour is governed by the crosslinking density and the stiffness of the segments in between the netpoints plays a minor role. According to our feeling this note holds only on traditionally cured EPs and not for those with co-networks.

This is the right place to emphasize that SMEPs have excellent durability, i.e., resistance to different environments, including high energy irradiation [71,72]. In a comprehensive review, Pretsch analysed the “functional determinants” of SMPs, including SMEPs [73].

### 2.2. Phase Separated Morphology

EPs are brittle materials due to their tightly crosslinked network. Their toughening has become a topic of research which is still ongoing [74]. One of the early toughening strategies was to generate rubber particles which are micron- or nano-scaled dispersed in the EP. These particles cavitate and participate in crack pinning. Moreover, they facilitate the shear deformation of the EP ligaments between the particles which is the major energy absorbing mechanism [75]. The in situ generated particles are from functionalized or non-functionalized rubbers, which are initially dissolved in the curable EP but segregate during the gelling/crosslinking of EP (called as reaction- or polymerization-induced phase separation). For EPs, the most powerful toughening agents are end-functionalized liquid nitrile rubbers. However, their use is accompanied with penalties especially in water uptake, stiffness and *T*_g_. Amine- and carboxyl-terminated acrylonitrile-butadiene rubbers (ATBN and CTBN, respectively) are preferred tougheners of EP. Note that their incorporation loosens and distort the network depending on the reactive or non-reactive nature of the modifiers. This, accompanied with increased ductility, should affect the SM behaviour of the corresponding EPs that has been already investigated. CTBN incorporation did not influence *R*_f_ and *R*_r_ (both remained close to 100%) but markedly increased the number thermomechanical cycles that could withstand the related system without failure [76,77]. Modification of EP with carboxyl-terminated PU yielded reduced *T*_g_ and enhanced ductility, similar to CTBN. The related blend exhibited excellent SM performance in fold-deploy tests [78]. Instead of making use of reaction-induced phase separation, toughened SMEPs can be produced also by incorporation of preformed rubbery nanoparticles. This concept was adopted by Wei et al. [79] who introduced crosslinked carboxylated nitrile rubber nanoparticles (50–100 nm) in up to 20 parts per hundred parts (phr) in EP resin. 

To avoid the disadvantages with rubber tougheners many works were dedicated to the toughening of EPs with amorphous or semicrystalline thermoplastics. Though their toughening mechanisms are essentially the same as for rubber modifiers, their incorporation is not associated with pronounced reductions in the basic mechanical and thermal properties. A great variety of amorphous and semicrystalline thermoplastic homo- and copolymers has been tried to modify EPs [74,80]. They can be dispersed via reaction-induced phase separation when soluble in the EP or its components, or mechanically when present as preformed particles, fibres. One of the preferred thermoplastic modifier is poly(ε-caprolactone) (PCL). PCL is a semicrystalline biodegradable polyester having a *T*_m_ of about 60 °C which makes it highly desirable as blend component and network constituent in various biodegradable SMPs [81]. PCL-modified EPs may show multishape and even multifunctional properties, as demonstrated later. Lützen et al. [82,83] produced a co-network containing cationically polymerized EP, covalently linked with hydroxyl-terminated semicrystalline PCL. Chemical reaction, though in small extent, occurred between the hydroxyl groups of PCL and epoxy groups of the EP resin. The PCL content was varied between 60 and 85 wt %. It has to born in mind that *T*_trans_ was linked with *T*_m_ of PCL in this EP/PCL system. *R*_f_ was 100% and changed marginally with the PCL content in fold-deploy tests opposed to *R*_r_ that decreased with increasing PCL content. In a follow-up paper the authors [84] compared the SM behaviour of cationically polymerized “partially crystalline EPs” containing up to 50 wt % two polyester diols, namely PCL and poly(ω-pentadecalactone), of similar molecular weights. Polymethyl methacrylate nanofiber, produced by electrospinning, was incorporated into DGEBA EP up to 0.2 wt % [85]. The results deduced from fold-deploy tests was that at low bending angles this EP exhibited excellent *R*_f_ and *R*_r_ values, while they were deteriorated at higher bending angles. Biodegradable SMEPs were produced from epoxidized soybean oil/PCL blends in which the PCL acted as “switching” component [86]. The authors found that the latent cationic curing agent did not supported the reaction between the epoxy groups of the oil and –OH groups of the PCL. Moreover, they concluded the presence of a semi-IPN (interpenetrating network) structure when PCL was present in 50 wt %.

Generation of interpenetrating networks (IPNs) is a further tool to tailor the properties (especially toughness and damping) of EP-based systems. In case of full IPN both resin components are crosslinked and each of them is present as a continuous phase. When between the crosslinked networks additional chemical bonds exits we speak about grafted IPN versions. The reason of mentioning full IPNs in this section is that they are also phase segregated though on nanoscale level [87]. EP-based IPNs, however, were not tested for SM properties. This is very surprising considering the fact that such full-IPN systems have a broad *T*_g_ range, sometimes even with two peaks due to incompatibility. Recall that the latter are key factors for triple-shape SMPs.

## 3. Multi-Shape Memory Epoxies

There are basically three possibilities to produce triple-shape SMEPs using EP resins: (i) exploitation of the “width” of the *T*_g_ range, (ii) generation of co-networks, semi-IPNs, full-IPNs and vitrimers with two “switching” temperatures and (iii) preparation of layered systems composed of two EPs with different *T*_g_s. It is noteworthy that they are still 1W versions.

The first approach assumes that the *T*_g_ range can be split into several sections in which a fraction of the segments is in the rubbery while the others are still in the glassy state. Basic prerequisite of multi-shaping is that the energy stored by deformation of the rubbery segments should be sufficient enough for shape fixing upon cooling and thus guaranteeing the recovery. According to the authors knowledge this has not yet proven for SMEPs. It is the right place to draw the attention to the fact that co-networks and especially full-IPNs, may possess very broad *T*_g_ ranges which would allow the required programing for triple-shape behaviour. Note that the onset and end temperatures of the *T*_g_ may be higher than 50 °C, as shown on the example of amine-cured EP/benzoxazine/reactive rubber blends [88].

**Figure 3:**
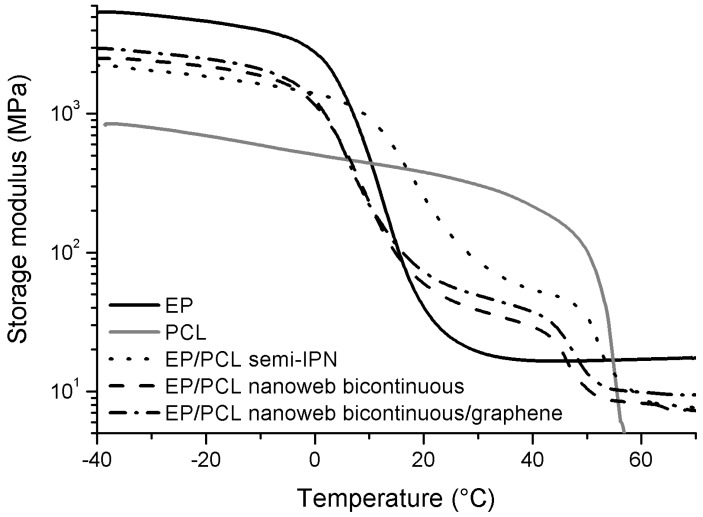
Storage modulus as a function of temperature for the EP/PCL with different structures and their plain constituents. Note: PCL content is 23 wt %.

The possibility of producing multi-shape structures making use of the thermally distinct plasticity and elasticity of vitrimers was nicely shown by the group of Xie [10], however, not on EP-based vitrimers. This is a task for the future. Semi-IPN or similarly structured EPs exhibited, however, triple-shape memory. Note that semi-IPN implies a bi- or co-continuous structure in which one of the phases is thermoplastic whereas the other is crosslinked thermoset. Luo and Mather [89] found an elegant way to trigger triple-shape memory in EP system. The authors embedded electrospun PCL nanoweb in a EP matrix. *T*_g_ of the EP, set by combination of aliphatic and aromatic versions, was below the *T*_m_ of PCL. Temperature of the EP curing occurred below *T*_m_ of PCL. Through infiltration of the PCL nanoweb by EP, a system of bi-continuous structure was formed. For programming of the two temporary shapes, set by different tensile deformations (second shaping at higher deformation than at the first one), *T*_m_ of PCL and *T*_g_ of EP were considered thereby choosing *T*_trans,high_ = 80 °C and *T*_trans,low_ = 40 °C, respectively. The EP/PCL system was tested for both dual and triple SM performances [89,90]. In the former case both *R*_f_ and *R*_r_ were close to 100%. During triple-shape tests the *R*_f_ and *R*_r_ data, measured for the second shape, were lower than for the first one. A similar approach was followed by Fejős et al. [91]. These authors incorporated also graphene into the PCL containing solution to be electrospun. This was aimed at facilitating the infiltration by EP *via* reinforcing the nanoweb. This study was aimed at checking whether the creation of a thermodynamically-induced co-continuous morphology yields comparable SM properties with that of the EP/electrospun PCL nanoweb system. The co-continuous structure in this case represents a semi-IPN (thermoplastic PCL/thermoset EP). This semi-IPN was achieved by dissolving the PCL (the amount of which agreed with that of the nanoweb in the reference system) in the EP followed by curing of the latter. The storage modulus—Temperature curves are essentially similar for the EP/PCL systems irrespective to their different structures (see Figure 3).

Triple-shape memory tests on EP/PCL systems were performed in a dynamic mechanical analyser at two different tensile strains thereby increasing the strain from the first to the second shaping. It was found that graphene incorporation into the PCL nanoweb negatively affected the *R*_f_ and *R*_r_ data. On the other hand, the *R*_f_ and *R*_r_ for both temporary shapes of the EP/PCL with semi-IPN structure were comparable with those of the EP/PCL nanoweb. Moreover, the semi-IPN structured EP/PCL outperformed the EP/PCL nanoweb with respect to *R*_f_ linked with the first temporary shape [91]. In a companion paper of the group of Mather [92] the above presented reaction- (or polymerization-)induced phase separation was exploited using amine cured EPs and PCL (added in 10 wt %). Using different EPs crystalline and amorphous EP networks were created. Accordingly, for one switch temperature (and thus for one intermediate shape) either the EP-related related *T*_m_ or *T*_g_, whereas for the other the *T*_m_ of PCL served.

Based on this result a bright future can be prophesied for the development of SMP systems with semi-IPN structure. This quote is supported by another aspect. Semi-IPN structured systems may overtake a further functional role, namely self-healing. This concept, i.e., combination of shape memory and self-healing, has been recommended by Karger-Kocsis as cited in Ref. [93]. In semi-IPNs the thermoplastic polymer (amorphous or semicrystalline) offers “switching” (SM) and “healing” (molecular entangling) effects, whereas the crosslinked thermoset acts for fixing of the permanent shape. It is intuitive that dissolving PCL in EP with increasing amount should change the reaction-induced phase separation morphology from dispersed, through semi IPN, to phase inverted one. On the other hand, the dispersion stage of PCL should influence the healing. This aspect will be discussed in detail later.

The feasibility of the “layering” strategy has been demonstrated by Xie at al [94]. They produced EP bilayers exhibiting triple shape memory performance. The bilayer system consisted of two EPs with different *T*_g_ values which were hold together by cocuring. The modulus-temperature trace of the bilayers showed two distinct *T*_g_s followed by two well developed rubbery plateaus. The two *T*_trans_ values, required for triple-shape performance, were selected from each rubbery plateau section. For the two temporary shapes the *R*_f_ values scattered between 71% and 97%, whereas the corresponding *R*_r_ values between 92 and 99%. These ranges depended on the relative thickness ratio of the bilayer constituents. This concept was followed also with EP nanocomposites containing nanosilica fillers [95].

## 4. Multi-Functional EP Formulations

It has to be underlined that SMPs are at the same time multifunctional materials since they may integrate multiple structural (high stiffness, strength, toughness) and structural (e.g., load bearing)/non-structural (e.g., sensing, actuation, self-healing, recyclability, biodegradability) functions [96]. The classification for multi-functionality also holds when the presence of properties, which are not linked with each other, is considered as criterion [14]. Based on the above introduced bilayer strategy with SMEPs the gecko adhesion (attachment/detachment to the surface) can be imitated, as demonstrated by the group of Xie [97,98]. The adhering, sticky layer of low *T*_g_ worked for adhesion, whereas the harder backing layer with higher *T*_g_ acted as SMEP actuator. In this case multi-functionality involves the adhesion to a substrate and the detachment of the adhesive upon demand (heating) enabled by the SM feature of the backing EP layer.

**Figure 4:**
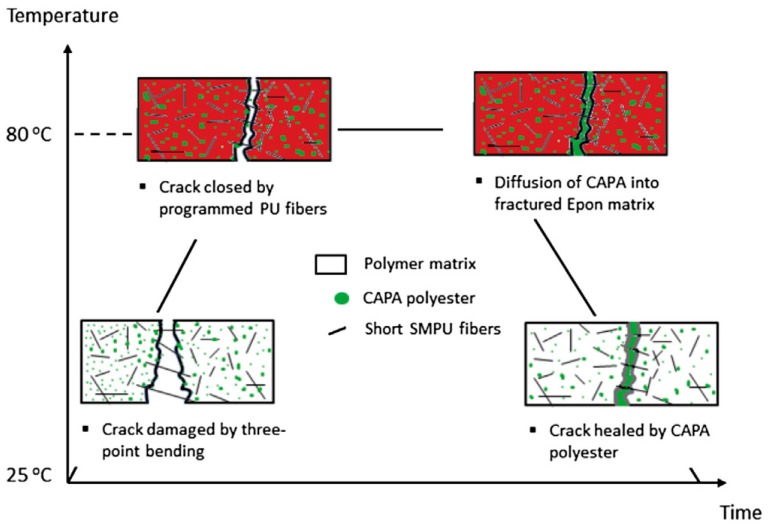
Schematic of the two-step healing process (i.e., close-then-heal) in a fractured short SMPU fibre-reinforced specimen ([102], reproduced with permission from Elsevier).

Combination of shape memory and self-healing was always a preferred topic. The initial concept was to close the crack prior to its healing. Kirkby et al. [99] inserted shape memory alloy (SMA) wires transverse to the crack direction in an EP specimen containing microencapsulated liquid healing agent (dicyclopentadiene) and dispersed Grubb’s first generation catalyst. It is noteworthy that the capsule-based healing strategy, being a version of the extrinsic healing possibilities [100], started with this healing agent and initiated a vigorous development [101]. The SMA wires did not break when the EP specimen fractured due to their higher ductility. The SMA wires were activated by direct current (dc) resulting in an axial recovery force that supported the crack closure ensuring the healing of the crack plane via polymerization of the dicyclopentadiene. SMA wires, however, can be replaced by suitable polymeric ones. Incorporation of thermoplastic micro- and nanofibers in various forms (short, mat) into EPs may contribute to shape-memory assisted self-healing. For that purpose, one has to find suitable polymers, polymer fibres, well adhering to the EP, which exhibit high ductility along with prominent strain hardening. The latter is usually accompanied with crystallization. To support the healing, however, a thermoplastic phase should be present in the healable EP. The feasibility of this concept has been shown by using short SM polyurethane (SMPU) fibres which were embedded in EP that contained a dispersed PCL phase (10 vol %)—see Figure 4 [102]. The lengths of the SMPU fibres were, however, above the critical length. In a companion paper of the same group effect of prestressing of endless SMPU fibres on repeated healing of EP/PCL (7 vol %) was investigated [103]. Strain hardening by cold drawing of SMPU fibres increased the healing efficiency which dropped, however, with increasing number of repeated healing.

The shape memory-assisted self-healing (SMASH, a term coined by the group of Mather [104]) was first presented on a phase-separated EP system containing electrospun nanoweb (random nanofiber orientation). Recall that this system is identical with the one used to demonstrate the triple-shape behaviour [89,90]. Self-healing was initiated by heating the damage area above both the *T*_g_ of EP and *T*_m_ of PCL. Crack closure was due to the strain energy release the EP matrix, while healing by molecular entangling of the molten PCL [104]. As demonstrated before, a suitable PCL dispersion in EP, produced via reaction-induced phase separation, may be as effective for self-healing as continuous PCL nanoweb. Wei et al. [105] studied the SMASH behaviour of EP/PCL blends whereby varying the PCL content between 0 and 23.3 wt %. The *T*_g_ of the EP matrix was above the *T*_m_ of PCL. PCL was present in dispersed particles up to 13.6 wt %, whereas phase inversion was concluded for 23.3 wt % PCL amount which was, however, not substantiated by dynamical mechanical analysis (DMA). For SM behaviour *T*_g_ of EP served, whereas for healing various temperatures between *T*_m_ and *T*_g_ were selected. The EP/PCL with the highest PCL content displayed the best healing performance (that should exhibit a semi-IPN structure according to our opinion). It is intuitive that in EP/PCL blends the morphology of PCL controls the healing which depends mostly but not exclusively, on the PCL amount. This aspect was studied in a recent paper of Karger-Kocsis [106]. In this work PCL was dissolved in 12.5, 25, 37.5 and 50 wt %, respectively, in amine-cured aromatic and aliphatic EPs cured with the same amine (Jeffamine D 230) to receive EPs with different *T*_g_s. *T*_g_ values of the parent EPs were lower (32 °C) and higher (90 °C) than *T*_m_ of PCL. The curing-induced phase separation morphology of PCL was studied by light microscopy. Additional information on the phase structure was deduced from DMA. Blending with PCL reduced the *T*_g_ of the corresponding EPs. Fully broken compact tension specimens were repeatedly healed at 80 °C which was close to or higher than the actual *T*_g_ of the EP/PCL blend. It was found that the transition of PCL from disperse to continuous phase depends not only on the PCL amount but also on the EP type and its curing. EP/PCL systems with semi-IPN structure exhibited markedly higher healing efficiencies compared to those in which PCL was present as disperse phase. The healing efficiency depended also on the temperature difference between the healing temperature and *T*_g_ of the EP with respect to *T*_m_ of PCL. Accordingly, also the segmental mobility within the crosslinked EP network is an important parameter for thermal mending.

Albeit in the above examples PCL worked as thermoplastic healing phase, other thermoplastic polymers may also be used. Wang et al. [107] for example used carnauba wax microparticles as healing agent in self-healing SMEP systems. *T*_m_ of the wax was beyond *T*_g_ of the SMEP. Note that wax is often used in phase change materials [108], so such systems may have a further functionality, namely thermal energy storage and release.

Intrinsic self-healing can be triggered via dynamic bonds (“reversible chemistry”), as well. Though the DA reaction is predestinated for this purpose, it is less explored for combination with shape memory in EPs. By contrast, pioneering works for the combination of SM and self-healing, are already available for the vitrimer chemistry. Lu et al. [109] cured a DGEBA-type EP with tricarballylic acid. Healing was achieved through transesterification at the fracture surfaces between two EP blocks in a confined space under action of compression stress recovery (constrained shape recovery). *T*_g_ of the EP was at about 60 °C and healing occurred in a compressed shape (<8.5%, eventually “cold”, i.e., below *T*_g_, programmed) between 120 and 150 °C for rather long time (18–52 h). The healing efficiency reached 60%. Attention should be paid to a further functionality of this EP, namely recyclability.

As mentioned in the introduction the actuality of this survey was also reasoned by novel preparation techniques. Electrospinning for example was not yet used for SMEPs. Zhang et al. [110] produced EP-based SM composite microfibers by means of coaxial electrospinning. Membranes composed of thermoset EP/PCL core/shell (*T*_g_ of EP below *T*_m_ of PCL) structured microfibers exhibited improved mechanical performance, good shape memory properties on both micro and macro levels (influencing the porosity of the membrane), thereby keeping good biocompatibility (owing to the PCL shell). This multifunctional membrane was foreseen as suitable candidate for tissue engineering applications.

## 5. Shape Memory EP Composites

Research on SMPs was extended for related composites from the early stage. This was fuelled mostly by two facts [111,112,113]: (i) need for other triggering mechanisms of the shape recovery than direct heating and (ii) great demand for SM polymeric systems featuring fast recovery along with high recovery forces. The latter is essential for actuators, which is favoured target application of SMP systems. Efforts to set the above properties using (nano)fillers and traditional reinforcements will be surveyed next.

### 5.1. Particulate-Filled

Micro- and nanoparticulates used in polymers are usually grouped according to their appearance: spherical (low aspect ratio), disc-like and fibrous (high aspect ratios). Next, we shall report following this grouping.

Yun and Liang [114] studied effects of carbon black (CB) microparticles (mean size 50 µm) on the *T*_g_ and stress relaxation behaviour of SMEP and the results were supported by an empirical rheological model. Nanoscaled CB, added up to 30 wt %—also with controlled local enrichment owing to a peculiar mould—enhanced the stiffness and worked for electrical conductivity in SMEP-based dry adhesive bilayer system [115]. The percolated CB network enabled to set the required *T*_trans_ (above the *T*_g_ of the EP) for temporary shaping via Joule heating under dc current. Since deformation-induced changes in a CB-filled SMEP affect the percolation network and thus its electrical conductivity, the deformation threshold should be determined [116].

Waterborne EPs were only recently used to prepare SMEP formulations. Major advantage of these environmental-friendly EPs is that they can be easily modified by water swellable and dispersible [117] or in situ produced (sol-gel chemistry) nanofillers. Dong et al. [118] followed the traditional silane-based chemistry to produce a silica sol that was mixed with the waterborne EP prior to its curing. The SMEPs with up to 2.5 wt % nanosilica showed good SM properties. *R*_f_ was enhanced by the presence of silica and both the *R*_f_ and *R*_r_ remained higher than 90% even after 10 thermomechanical (tensile mode) cycles.

The group of Gall [119,120,121] checked the SM properties of EPs containing up to 40 wt % SiC of different (300 and 700 nm) mean sizes. Temporary shape was set both above and below that of the *T*_g_ of the related EP in flexure. The nanocomposite generated higher recovery stress than the unfilled matrix. At 40 wt % SiC loading permanent strain was noticed. It is noteworthy that SiC is a very efficient filler to enhance the thermal conductivity of the corresponding matrix. This is a key issue for thermally-activated SMPs. However, direct heating is not always practical and thus researchers were looking for its alternatives. Indirect heating can be achieved for example by adding infrared absorbing, electrical (Joule heating) and magnetic conductive (inductive, hysteretic heating) fillers. Some of them, such as infrared irradiation, absorption of radiofrequency energy, may result in wireless, remote actuation of SMPs. Hazelton et al. [122] showed the feasibility of radiofrequency actuation of EP containing magnetoelectroelastic particles, added in ≤15 vol %. Surface modification of nanofillers may be also a tool of tuning the SM properties. Iijima and co-worker [123] modified the surface of TiO_2_ nanoparticles with an anionic surfactant having a polyethylene glycol (PEG) chain, large enough for crystallization. *T*_m_ of PEG of these “PEGylated” TiO_2_ particles could be used as *T*_trans_ when embedded in an EP matrix in ≤13 wt % amount.

Organophilic clays of disc-like structure have been incorporated in EP systems in order to improve their (fracture) mechanical performance. Their effect has been checked also with respect to SM. It was found that organoclay in 3 wt % enhanced the recovery speed without reducing *R*_r_ [124]. Beloshenko et al. [125,126] added thermoexpanded graphite (which belongs to the disc-like fillers, too) with and without kaolin microparticles in EP and studied the SM properties in uniaxial compression. Though no Joule heating was found, meaning that the material remained insulator, the resistivity of the composites increased steeply at the *T*_g_ of the resin. Graphite/graphene derivatives are also explored as nanofillers in SMEPs. Zhao et al. [127,128] investigated the mechanical properties and SM effects of graphene nanoplatelets (up to 2 wt %) in EPs and found better SM performance and enhanced recovery stress and speed compared to the neat EP especially at 1 wt % graphene content. Thermally reduced graphene oxide, introduced up to 3 wt %, was also tried as modifier of SMEP [129]. *R*_f_ increased with the modifier content reaching almost 100% in fold-deploy test. The shape recovery was the fastest for the SMEP with 1 wt % thermally reduced graphene oxide.

Among the novel carbonaceous nanofillers carbon nanotubes (CNTs) and nanofibers (CNFs) received the greatest attention in SMP research. Li et al. [130] incorporated multiwall CNT (MWCNT) up to 0.83 wt % in an aromatic diamine cured EP and studied the SM behaviour of the corresponding EP nanocomposites in compression upon different programing protocols. According to their results *R*_f_ and *R*_r_ were governed only by the deformation temperature while the constrained recovery properties by both the deformation temperature and MWCNT content. Effect of MWCNT reinforcement appeared most pronounced when the deformation temperature agreed with that of the *T*_g_ of the SMEP composite. SMEP composites containing CNT (up to 10 wt %) and boron nitride (BN, kept at 4 wt %) were produced and tested by Lu et al. [131]. The research strategy with this nanofiller combination was to enhance the infrared light absorption (*via* CNT) and heat conductivity (*via* BN). The temporary shape of the specimens was fixed by the traditional way, for its recovery, however, infrared irradiation was used. CNT at 1 wt % was incorporated in a polyamine cured waterborne EP and the SM properties tested under isostress and isostrain conditions using a thermomechanical analyser (TMA) device [132]. Note that TMA is rarely used by contrast to DMA for SM testing. The corresponding EP exhibited a very broad *T*_g_ range allowing the authors to investigate the SM behaviour below and above the *T*_g_ at different temperatures. Interestingly, the *R*_f_ and *R*_r_ ratios were almost 100% even when the shaping occurred below *T*_g_. In order to avoid the agglomeration of CNT, Dong et al. [133] have chosen an interesting strategy. EP was grafted first with a nonionic surfactant, viz. polyoxyethylene octylphenyl ether, using toluene diisocyanate. This compound worked as reactive emulsifier in waterborne EP/CNT. Moreover, its presence was manifested in a further reversible *T*_g_ transition (assigned to dangling chains), superimposed to that of the EP matrix. This could be used as a second switch temperature in programing to achieve triple-shape behaviour. Reversible plasticity SM effect in EP with and without up to 2 phr MWCNT was studied and modelled by Abishera et al. [134,135]. In this work, the temporary shape was fixed by plastically deforming the material at a temperature lower than its *T*_g_. MWCNT improved this kind of SM behaviour in a great extent.

Lu et al. [136] combined electrical conductive carbon nanofiber (CNF) with electromagnetic Ni nanostrands to render the initially insulating EP to electric conductive. The resulting SMEP was activated by electrical resistive heating (Joule effect) and displayed very fast recovery. This was supported also by the high thermal conductivity, being a “by-product” of the filler combination used. Untreated and silanized vapour-grown CNF was incorporated in EP to improve its SM performance [137]. Silane surface treatment served for a better dispersion of CNF in the EP matrix. CNF presence caused a significant change in the *T*_g_ of the EP because the silane contained –NH_2_ functional groups, reactive with the EP. The shape recovery of the EP was improved by CNF dosage and the recovery ratio was further enhanced when silanized CNF replaced the untreated one in the corresponding formulation. SMEP nanocomposites were produced by hot pressing of a freeze-dried compound composed of amine-cured waterborne EP, vapour-grown CNF [138,139] and/or in situ produced silica (organosilane-based sol-gel technique) [138]. The SM behaviour of these nanocomposites containing up to 2.5 wt % nanofiller was investigated in fold-deploy tests at temperatures close and slightly above the *T*_g_ and excellent *R*_f_ and *R*_r_ values were obtained.

CNFs are prone to self-assembly and the related structure can be deposited in form of nanoweb (buckypaper). This buckypaper is not only an efficient reinforcement but enables the electro-activated triggering (Joule heating) of SM at the same time. This possibility was explored by the group of Leng [140,141,142] who coated or impregnated the buckypaper with SMEP. In order to improve the heat conductivity of the buckypaper or the corresponding nanocomposites BN was also incorporated or blended into the EP, respectively. Nanoweb (nanopaper) can also be produced using reduced graphene oxide. When it is impregnated by an SMEP the electric resistive heating of this graphene oxide paper, possessing excellent heat conductivity, may overtake the trigger function for SM effect. This was demonstrated by the group of Bhattacharyya recently [143].

Remote controlled multishape SMEP nanocomposites were produced by the combined use of Fe_3_O_4_ nanoparticles and CNT whereby exploiting the fact that these additives work at heat sources at very different radiofrequency ranges (296 kHz and 13.56 MHz, respectively) [144]. The dual shape performance of this magnetically-responsive SMEP containing 5 wt % Fe_3_O_4_ and 0.4 wt % CNT was checked by the traditional way. Multishape behaviour could be generated in a thermally-induced temporary structure parts of which contained selectively Fe_3_O_4_ particles or CNT after subjecting to exposure at 296 kHz (recovery of the Fe_3_O_4_ SMEP region) and subsequently at 13.56 MHz (recovery of the CNT SMEP region).

### 5.2. Fibre- and Fabric-Reinforced

As reinforcements in EP-based composites various fibre assemblies are traditionally used. Their range covers nonwoven mats, different woven structures and fabrics with unidirectional (UD) fibre alignment. UD fibres are usually present in prepregs from which advanced composite laminates with various lay-ups are produced. Nowadays, UD fibre-containing fabrics, even composed of two or more different materials (hybrid reinforcement), are marketed. The reinforcing fibres may be of synthetic (glass, carbon, aramide) or of natural origin (mineral fibres such as basalt, plant fibres such as flax, jute, sisal and the like). The stiffness and strength of the reinforced EPs are prominently higher than those of the matrix, moreover, they can be tuned upon request by different ways (e.g., type and amount of the reinforcement, fabric layering and orientation). High stiffness and strength are desirable properties for the recovery of SMEP composites in order to compete in some applications with SMAs. Therefore, great efforts are undertaken to prepare SM polymeric composites with enhanced recovery stress and reduced recovery time. Unfortunately, reinforcing with fibrous structures, especially when present in high amount in the related composite, reduces the ductility and deformability thereby restricting the design freedom for temporary shaping. So, improved recovery stress is usually achieved at the expense of the shaping deformability (see Figure 1).

In some cases, for example during bending of UD-fibre and fabric containing laminates, limits in the deformation are broadened via a peculiar deformation of the fibres [145]. They undergo microbuckling in the compressed zone of the specimen or part thereby realizing relatively high deformations [146,147]. Accordingly, an advanced EP-based composite with high UD carbon fibre (CF) content may exhibit at about 5% nominal bending strain, though the ultimate strain of the CF is less than 1% [147]. To make use of this microbuckling, however, attention should be paid on the right selection of the matrix, fibre/matrix adhesion and adjustment of the shape programming procedure to the composite’s properties [145]. Basit et al. [148,149,150] prepared layered composites with symmetric and asymmetric hybrid reinforcements and studied their SM properties in bending mode both in unconstrained and constrained conditions in cyclic tests. Note that unconstrained testing is accompanied with stress free deformation and thus suited to determine *R*_f_. By contrast, constrained deformation is performed under load based on which the recovery stress can be measured. The cited authors made use of the Joule heating, produced via UD CFs embedded in the mid-section (neutral axis) of the specimens, to set *T*_trans_ for temporary shaping and to get those temperatures where the shape recovery was investigated. This system worked basically as an actuator. Progress of the recovery stress was studied as a function of *T*_trans_ and recovery temperature.

There are further possibilities to widen the shaping freedom with SMEP composites, more exactly with SMEP-containing structural parts, such as beams [151], hinges and booms [152], self-deployable structures [153,154] where design aspects dominate. Positioning of the reinforcing layers and selecting their types may affect the deformability, as well. Just one example to underline this claim: a fabric with the same pattern and surface weight has completely other deformability when produced from stiff CFs instead of more “compliant” natural fibres (NF).

**Figure 5:**
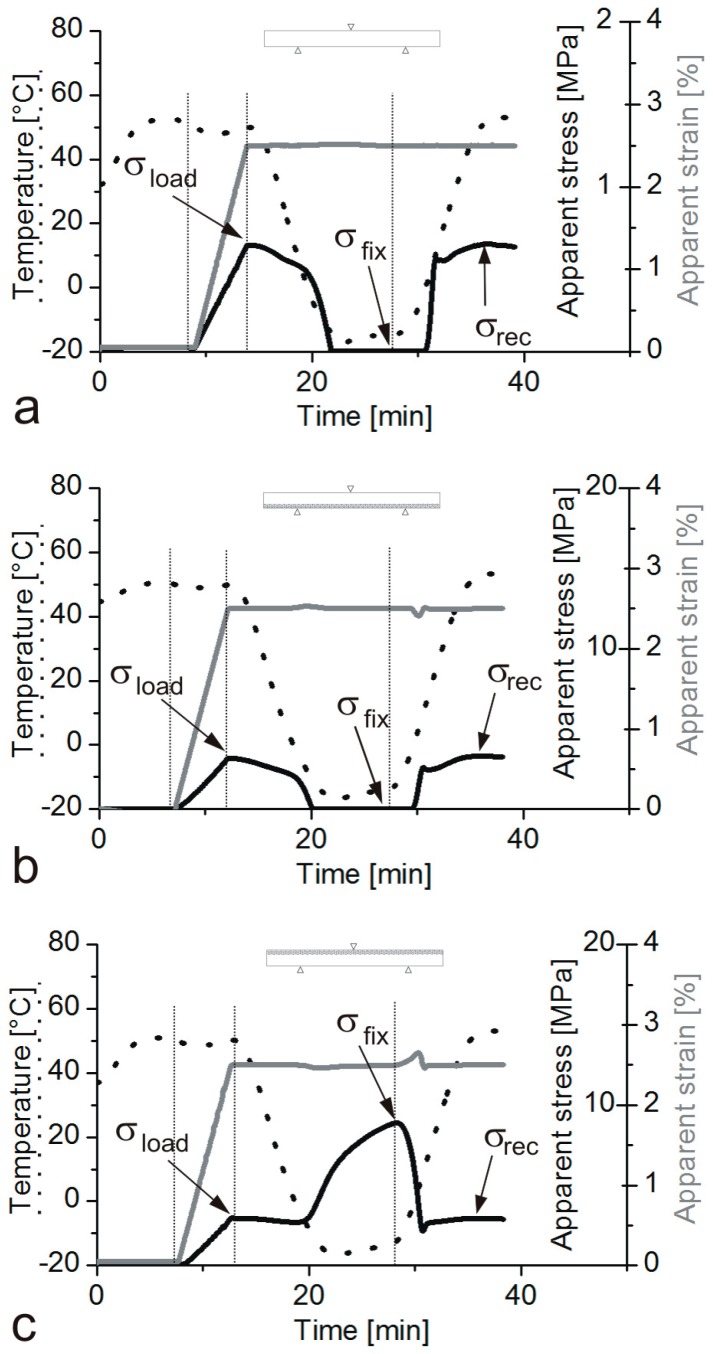
Shaping and unconstrained recovery of the neat EP (**a**) and its asymmetrically positioned CF-fabric (**b**,**c**) reinforced composites (containing two CF-fabric layers in the bottom and in the top, respectively) in bending (based on Ref. [156], reproduced with permission from BME-PT).

Four glass fibre (GF) fabric layers were infiltrated by EP yielding a composite with rather high reinforcement content (38 vol %). It was found that with increasing GF fabric content *R*_f_ decreased opposed to the recovery stress that was enhanced by one order of magnitude (ca. 40 MPa). Parallel to that the critical bending strain, not causing observable damage, was highly reduced compared to neat EP (from >6% to 1%) [155]. Guo et al. [47] incorporated one single GF fabric layer with and without additional nanosilica coating in a liquid crystalline EPs. *R*_f_ was improved by this surface treatment of the GF fabric without affecting the *R*_r_ values. The SM behaviour of this SMEP composite was not influenced by the chemical structure of the liquid crystalline EPs. Fejős and Karger-Kocsis [156] studied the SM properties of CF fabric reinforced composites in bending. The CF layers were either on the tensile or on the compression side of the specimens during unconstrained and constrained tests. Again, the critical bending strain for SM testing was highly reduced by the reinforcement. The temperature and stress developments as a function of time are depicted in Figure 5 for the EP and EP/CF composites with CF layers positioned on the top (compression side) and bottom (tensile side), respectively. One can notice, that the stress values needed for deformation and measured during recovery agree well with each other. This implies good *R*_f_ and *R*_r_ data which were >93%, in fact. For the shape fixing required stress of the specimen containing CF layers on the top (under compression) was, however, much higher than the recovered one. This suggests the onset of microbuckling associated with an increment in the elastically stored energy. A substantial part of this is released, however, before passing the *T*_g_ of the EP matrix—see the courses of apparent stress and temperature in the related time interval in Figure 5.

This finding suggests that reinforcing layers of such SM composites which are designed to deliver high recovery stress should stay under compression and they recovery should be triggered at the onset of *T*_g_ or below. Recall that this recommendation is in full agreement with that of gained from studies on SMEP composites [146,147,157].

**Figure 6:**
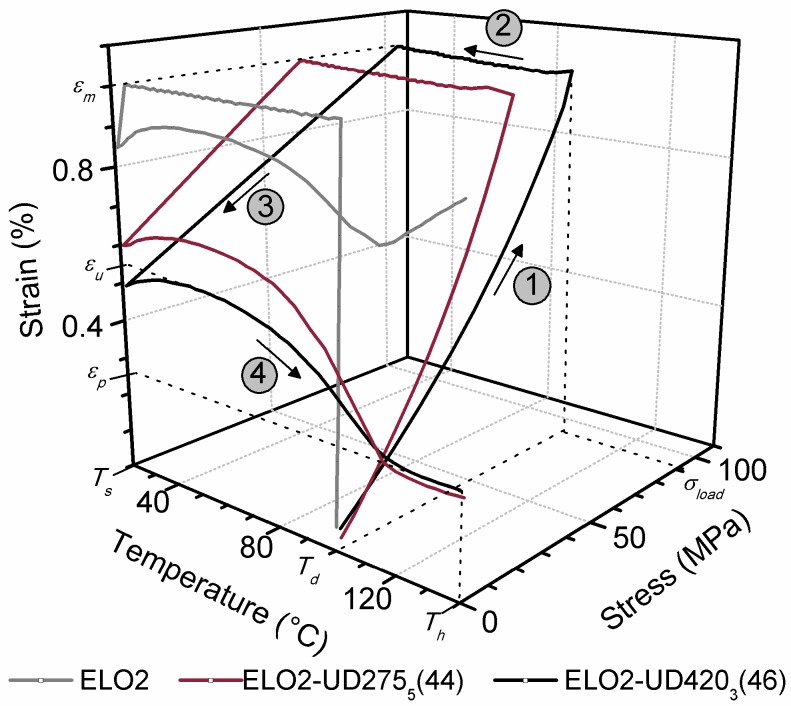
3D representation of the shape memory behaviour of the epoxidized linseed oil-based bioresin (ELO2, *T*_g_ ≈ 90 °C) and its UD-flax reinforced composites. Designation: the numbers indicate the steps of shape memory test: (**1**) deforming at *T* = 100 °C to ε_m_ = 1%, (**2**) shape fixing by cooling to *T* = 20 °C while keeping ε_m_ = 1%, (**3**) unloading, (**4**) stress-free shape recovery. Notes: UD-reinforcements had different surface weights (275 and 420 g/m^2^, respectively, due to fine and coarse flax yarns) but their overall content (44 and 46 wt %) was comparable owing to the number of layers (5 and 3, respectively) built-in.

Following the actual research trend, attempt was made to produce SMEP composites from fully renewable resources. To produce a “biocomposite”, epoxidized linseed oil, cured by anhydride, was selected as matrix and flax fabrics (nonwoven mat, twill-weave and quasi UD fabric) served for reinforcement [158]. Recall that NF may be a favoured reinforcement for SMEP because its presence imparts the deformability of the corresponding composite in lesser extent than traditional reinforcing fibres. The flax fibre content (<58 wt %) was varied through the number of fabric layers, their surface weight and fineness of the flax fibres. The *R*_f_ and *R*_r_ data of this epoxidized linseed oil based EP were 92 and 25%, respectively. The very low *R*_r_ substantiates that the recovery performance is controlled by the crosslink density, which was much lower in this “bioresin” than in a typical “petro-based” EP. *R*_f_ was reduced and *R*_r_ improved by the flax reinforcement, though they remained still moderately low [158] (see Figure 6).

For preparation of SMEP composites with electrically triggered (Joule heating) shape recovery CF mats with and without further modifications are mostly used. Composite preparation occurs usually via resin infiltration, resin transfer moulding (RTM). Modification of the CF mat may target different tasks, such as better bonding to the matrix, tailored electric conductivity, improved thermal conductivity, temperature sensing. Silver nanoparticle decorated reduced graphene oxide was placed on CF mat prior to EP infiltration [159]. The electrical conductivity of the SMEP composite was markedly improved by the Ag nanoparticles. For the electroactivation of the shape recovery the resistive Joule heating at low voltage (<10 V) was enough—supported also by the homogeneous temperature distribution within the composites. In a follow-up paper by the group of Lu [160], the CFs of the mat were oxidized. By this way, the bonding between the reduced graphene oxide and CF was enhanced resulting in improved reinforcement of the SMEP through van der Waals forces and covalent bonding. In a further paper [161] aluminium nanopowder/-layer was placed onto the CF mat *via* sputtering in vacuo followed by its silanization. The electrical resistivity of the corresponding SMEP composite could be tuned by the layer thickness of the siloxane-grafted Al layer. This improved the electrothermal efficiency of the shape recovery process. In a recent paper thermochromic microcapsules were incorporated into the SMEP before infusing the reinforcing CF mat [162]. These thermochromic particles worked for temperature sensing of the thermal/electrical actuation process of the corresponding SMEP composite.

Though there is a vast knowledge on SMEP-based composites it is still partly used for optimizing their SM possibilities. This note holds for reinforcement-related issues and even for the loading mode adapted for temporary shaping. For example, no report is available on torsion loaded 3D braided textile-reinforced systems which should exhibit very high recovery forces. A further aspect to which attention should be paid is the durability of SMEP nano- and macrocomposites. Works have already been focused on how the SM performance of SMEP composites is changing owing to exposure of different environments [73,163,164].

## 6. Shape Memory EP Foams

SMEP foams display a unique profile of low density, high compressibility and SM feature. SM may be eventually combined with other functions (e.g., self-healing, sensing) and properties (auxetic foam characterized by a negative Poisson ratio [165]). Foaming itself is a further tool to tailor the foam properties for application requirements. Foams have markedly lower mechanical stiffness and strength than the corresponding bulk materials but possess high compressibility and different Poisson effects. Foams are classified according to their cell structure: (i) open cell (permeable), (ii) closed cell (impermeable) and (iii) coexistence of open and closed cells. On the other hand, practically all foams show a similar course when their compression stress-strain curves are considered. In the initial stage the stress is increasing owing to axial deformation and bending of the cell wall struts. This is followed by a small increase in the compression stress upon further deformation. In this plateau-like region, the cell struts buckle massively. Final failure is preceded by considerable strain-hardening during which the compressive stress steeply rises due to collapse of the cells (densification). The above scenario already suggests that there is compression strain limit for SM that should not be surpassed to achieve efficient shape recovery. The above deformation modes also suggest that to tune the mechanical and SM performance, the cell structure via its struts should be reinforced. Needless to say that for this purpose nanoparticles seem to be the right choice. Although majority of the SMP foam related works was dealing with PU foams [166,167,168], SMEP foams became also under spot of interest owing to their excellent environmental durability, including space conditions [166]. Recall that space use is favoured by the fact that the SMEP foam expands from a packed shape to a much larger one with minimal shrinkage in direction perpendicular to the expansion (small Poisson effect). Foaming of EPs is, however, not a simply task and this is the major reason why only few related works are available. EP foams, usually produced by proprietary technologies, with various relative densities (density reduction in percentage normalized to that of the bulk EP) are on the market [167]. The SM behaviour EP foams is characterized according to the protocols developed for 1W dual-shape SMPs. Accordingly, the SMEP foam is compressed until a given strain at temperatures slightly below or above the *T*_g_, then this temporary shape is fixed by cooling below *T*_g_ and kept there eventually for longer time (“cold hibernated elastic memory”). The permanent shape is regained in expansion at temperatures beyond *T*_g_ [167,168]. This programing/recovery processes are often repeated and the results of these cyclic tests are sometimes referred to durability. Di Prima et al. [169] quoted that SMEP (relative density 16%) is capable of recovery from compressive strains up to 90%. This can be considered as the maximum strain threshold. The optimum temperature for compressive “packing” of the foam (*T*_trans_) was slightly below *T*_g_. In a companion work [170] the cyclic compression behaviour (prestrain and hold time varied) SMEP foams with relative densities of 20%, 30% and 40%, respectively, was studied. The deformation was visualized by micro-computed tomography based on which a damage threshold was substantiated. The authors found that the “packing” temperature had negligible effect on the unconstrained recovery but a significant change was observed in constrained conditions. Vialle et al. [171] demonstrated that thermal activation of shape recovery of SME foam can be triggered by remote activation. For that purpose, nanomagnetite particles, working as magnetic susceptors, were incorporated into the foam up to 10 wt % without sacrificing the thermomechanical properties of the foam.

New impetus to the SMEP foam research was given by the development of the solid state foaming [168,172]. In this procedure a solid tablet which contains the EP, its hardener and other additives is overheated generating foaming associated with the curing of the EP. EP foams—also with clay nanofiller [173]—produced by this technique have been investigated for SM using different loadings (compression, torsion, bending) and environments (“ground” and microgravity or space) [166,174].

A further leap in the SMEP foam research was the adaption of the latex technology developed by the group of Fu [175,176]. In this procedure the composition consist of a waterborne EP, a reactive EP-grafted nonionic emulsifier (the same as reported in [133]), a room-temperature curing agent, a physical blowing agent (alcohol or water) and eventual further nanoadditives (CNF, in situ produced silica). The related mixtures were freeze-died and their foaming occurred in vacuo at *T* = 100 °C. Incorporation of vapour-grown CNF up to 1 wt % enhanced the mechanical strength and electrical conductivity (the percolation threshold was found at 0.6 wt %) of the SMEP nanocomposite foam. *R*_f_ was marginally increased in the presence of CNF compared to the unmodified SMEP foam (scatter range 94–95%) and its value did not change as a function of the thermomechanical cycling (i.e., SM programing/recovery in compression mode up to 80% strain). By contrast, *R*_r_ increased from 95% to 98% in the first cycle when CNF was incorporated. Both foams had *R*_r_ values higher than 98% after the second cycle. *R*_r_ tended to reach 99% with increasing cycle number that was attributed to a so called “training effect.” In a further paper [176] nanosilica, produced in situ making use of the silane based sol-gel chemistry, served as nanofiller in the cell struts of the foam. In this work, the residual water after freeze-drying fulfilled the role of physical blowing agent. Therefore, the tests were aimed at studying the effects of drying time. SM behaviour was investigated in cycle tests setting a compression strain of 70%. Good *R*_f_ and excellent *R*_r_ values were measured; *R*_f_ was markedly enhanced after the second cycle and in the first one a strong effect of the actual water content was found. *R*_r_ was not influenced either by the cycles or the residual water content after freeze-drying (that was not quantified).

SMEP foams may be also multifunctional materials. Li and Nettles [177] reported on the preparation of a self-repairing thermoset syntactic foam. Note that in syntactic foams hollow particles (usually glass beads) are incorporated into the resin. Crack closure was achieved when the cracked specimen in “working shape” (temporary shape produced with confinement above and cooled afterward below *T*_g_) is reheated above *T*_g_ while confinement is held, followed by cooling below *T*_g_. The crack closure may be combined with full healing making use of intrinsic and extrinsic healing possibilities, as introduced above in Section 4.

## 7. Shape Memory EP/Shape Memory Alloy (SMA) Combinations 

**Figure 7:**
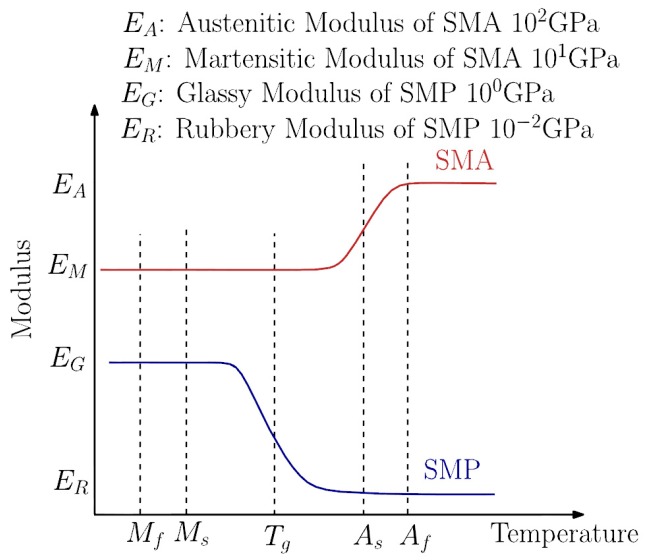
Modulus as a function of temperature for SMP and SMA, schematically. Note: this figure emphasizes the effect of the corresponding transformation (“switching”) temperatures ([178], reproduced with permission from Elsevier).

Shape memory alloy (SMA) was discovered in 1932 but its importance was recognized in 1962 after the development of NiTi-alloy. NiTi-alloy is preferred over iron- and copper-based SMAs showing better phase stability and thermomechanical performance. Similarly, to SMPs, SMAs can return to their original shape when subjected to memorization between two transformation phases. The trigger function of the SM effect is the temperature (direct, indirect) or magnetic field. SMAs may exist in three phases: twinned and detwinned martensitic structures which are stable at lower temperatures and austenite that is stable at higher temperature. When SMA is heated, the phase transition from martensite into austenite starts. The austenite start-temperature (*A*_s_) linked with the onset of this transition that is completed at *A*_f_ (finish temperature) (see Figure 7). During this process, the SMA is contracting, also under load, thereby recovering its original shape. During cooling the transformation starts to revert at *M*_s_ (starting temperature of the martensite transformation) and it is finished at *M*_f_ [178,179] (see Figure 7). It has to be underlined that this martensite-austenite transformation is associated with considerable “hardening” (increase in stiffness, modulus) which is opposed to what EP experiences at its *T*_g_ (see Figure 7).

Figure 7 already suggest that in order to combine the performances of SMA and SMEP [180], the *T*_g_ of the latter should lay between *M*_s_ and *A*_s_. It should be born in mind that the modulus of SMA in either of its phases is higher by two orders of magnitude than that of the EP. This large difference in the moduli implies that an optimum content of SMA exist for the corresponding smart composite otherwise the SMA feature dominates [178]. There is a further characteristic temperature, viz, *M*_d_, above which martensite can no longer be stress-generated. If an SMA is stressed between *A*_f_ and *M*_d_ a detwinned martensitic structure forms. *A*_s_ austenite is the thermally stable phase in this temperature range under stress-free condition, the SMA “springs back”—without thermal activation—when the stress is no longer applied. This peculiar elasticity is also called pseudoelasticity or (transformational) superelasticity. So, SMAs exhibit SM effect (1W or 2W) and pseudoelasticity. In case of 1W SM the SMA retains its deformation after removal of the external load and recovers its original shape upon heating. In the case of 2W SM the SMA can remember its shapes at both high and low temperatures. In order to achieve 2W SM, however, it should be “trained” during which the maximum recovery strain is highly reduced owing to permanent defects introduced in the structure. Reversible shape changes occurs in this case by applying suitable temperatures without external load [179]. R & D works tried to make use of the pseudoelasticity and/or SM effect of SMA when incorporated into SMEP hybrid composites. The key requirement of exploiting the above SMA-related characteristics is, however, a good bonding between the SMA (generally having an oxidized surface) and EP [181,182]. To improve the interfacial adhesion between SMA (wire, foil, band) and EP various strategies were followed such as SMA coupling with reactive (epoxy functionalized) silane [183], roughening of the SMA surface by physical and chemical methods [181]. The photoelasticitic feature (birefringence) of adequate EPs may be very helpful to study the adhesion of SMA to EP [184].

Under spotlight of the “SMA/EP hybridization” research was initially the pseudoelastic behaviour of SMA. Advanced composites’ laminates, composed of UD fibre reinforced EP prepregs, have excellent in-plane properties in contrast to moderate out-of-plane ones. Out-of-plane type loading, which occurs under transverse subcritical (i.e., no perforation) impacts, eventually repeatedly, causes fibre matrix debonding with massive matrix cracking and delamination between the adjacent prepreg layers. This strongly deteriorates the in-plane measurable properties. That is the reason why the damage tolerance of advanced composites is usually characterized by compression after impact testing. Tsoi et al. [185] incorporated SMA wires with and without prestretching (superelastic NiTi in austenitic state, martensitic NiTi- and NiTiCu-based wires and for reference stainless steel wires) in EP/UD-GF composites with various prepreg lay-ups and subjected them to subcritical impact. The working hypothesis was that the impact energy will also be absorbed by the superelastic wires transforming them from austenite to martensite. In case of the martensitic wires most likely their effect on the internal stress state of the composites was the research target (explained below), though not mentioned explicitly. After impact, the energy is released again by the reverse transformation. The damage development was assessed by ultrasonic C-scanning. It was found that beneficial effects of the SMA “wiring” appeared mostly at higher impact energies. At lower impact energies, the damage area was comparable with that of the reference composite. Prestretching, density (fibre volume fraction) and positioning (preferentially at the bottom of the laminate plates where tensile stresses prevail) of the superelastic wires were found as key parameters, too [185]. Similar results were reported also by Sun et al. [186]. Note that the thermally triggered SM effect (1W SM) of SMA may also contribute to damage tolerance and vibration damping of advanced composites. In this case, the prestressed SMA wires, embedded in laminate composites, undergo the phase transformation from martensite to austenite upon curing of the composite and tend to contract. Recall that for triggering this transformation, *A*_f_ should be surpassed in the curing cycle. If the contraction is prevented through the good adhesion to the matrix, then positive recovery stresses arise which improve the resistance to damage under static, cyclic [187] and dynamic loadings of the coupons or structures. This was demonstrated in a model experiment by Khalili et al. [188]. The contraction associated with the martensite to austenite transformation upon heating along with the related large recovery stress were used in the former introduced “close and heal” self-healing concepts [99,189]. Note that this transition was thermally induced via Joule heating of the electric current. The temperature development in the SMA wire, embedded in the EP, was also investigated and modelled [190].

Efforts were also undertaken to make use of the martensite/austenite phase transformation of SMA particles and discontinuous fibres. Zhang et al. [191] found that addition of 3.5 wt % SMA filler in EP (resulting in a two-layer structure owing the segregation of SMA) prominently increased the stiffness (modulus), especially in the *T*_g_ range laying beyond that of *A*_f_. The numerical model of Pulla et al. [192] confirmed that the compression stress-strain behaviour is improved in presence of SMA particles (up to 50 wt %) when they underwent the martensite/austenite transformation. Modelling may deliver valuable advices on how to prepare hybrid EP composites with a “functionality-tailored” morphology. Jiang and Batra [193] for example gave recommendations for the adequate incorporation of piezoelectric (small strain but quick response to electric field) and SMA inclusions (large strain but slow response to thermoelectrical triggering) based on a micromechanical model. The magneto-mechanical actuation possibility of EP/ferromagnetic SMA single crystal composites was also modelled and experimentally validated by Glock et al. [194]. According to the model prediction the EP matrix should have very low elastic modulus in order to reach large strains with this hybrid.

Low elastic modulus of the composite, at least at the folding, may be a requirement of self-deployable (space) structures when deploying is activated by SMA wires in a 1W SM process. This problem was solved by Todoroki et al. [195] who produced a partially flexible CF-EP laminate. “Partial flexibility” was guaranteed by a silicon rubber section between EP-based ones which all infused the same CF reinforcement. Discontinuous SMA wires were inserted in the silicon layer but with large enough protrusions into the EP section. This partially flexible composite was bent by the plastic deformation of the SMA wires and its recovery, associated with phase transformation, triggered electrically by Joule heating.

The vibration characteristics of EP composite plates can be tuned by the incorporation of SMA wires and fabrics that was shown by Zhang et al. [196]. The authors concluded that changes in the vibration properties are less significant at low temperatures but it is sensitive at high temperatures owing to the martensite/austenite phase transformation. The natural frequency of the composite beams depended not only on the temperature but also on the SMA content (volume fraction). EP/SMA hybrids may work, however, also in the opposite direction to vibration damping, i.e., toward energy harvesting, as demonstrated by Lu et al. [197].

**Figure 8:**
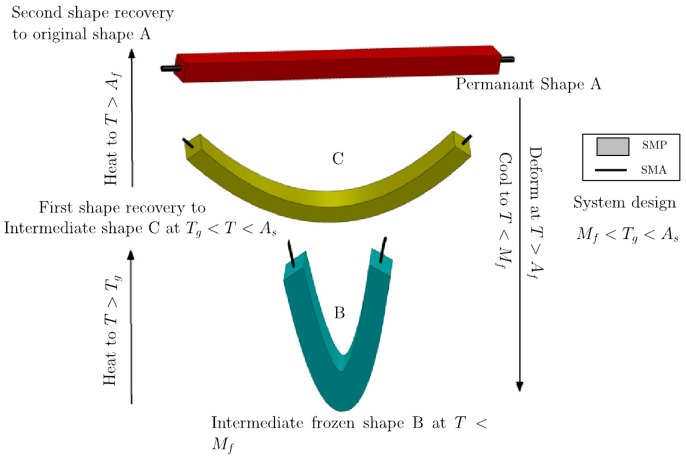
Three-state configuration (triple-shape memory) obtained by SMEP and SMA wire ([178], reproduced with permission from Elsevier). Note: for designations see Figure 7.

Hybridization of SMEP with SMA wire is an interesting tool also to produce triple-shape memory composites as shown in Figure 8. [178]. Prerequisite of this kind of SM behaviour is that *T*_g_ of SMEP is between *M*_s_ and *A*_s_. Ghosh et al. [178] in their work also disclosed how to produce a triple-shape composite element with discontinuous SMP matrix and continuous SMA wire reinforcement.

It is worth of noting that changes in the electric resistivity, with and without phase transformation, of the SMA can be used for structural health monitoring because SMA may work as a strain-sensing element. Mapping of the Joule heat caused temperature field may be useful to localize the damage [198]. Note that structural health monitoring of composites is the most actual challenge [199].

## 8. Two-Way Shape Memory EP Systems

2W SMPs change their dimension upon suitable stimulation without the requirement of any preformation. 2W SM feature was shown on the examples of hydrogels (thermally reversible order/disorder transition) [200], liquid crystalline elastomers (thermally reversible nematic/isotropic transition) [7,13,16,111,200], polymers with one or two semicrystalline phases under constant stress (thermally reversible melting/crystallization) [7,13,16,84]. Note that constraint conditions may be generated also by chemical/morphological architecture [201,202]. Though any of the above approaches was adapted for EPs, some of them may work for 2 W SM (e.g., (semi)-IPN structuring, liquid crystallinity).

On the other hand, there are several possibilities to introduce a reversible stress state, accompanied with shape alterations, in plain EPs and EP-based systems. They may be termed as mechano-responsive SMPs albeit the trigger function is not external load but usually heat as shown later. The bilayer (lamination) concept, credited to Langer and Lendlein [203], makes use of the mismatch of the thermomechanical properties of the laminated layers thereby generating flexural stress. This bilayer concept was proven for 2W SM effect using plain EPs and EP/EP-matrix-based composites, as well [7,16,203]. In a recent paper Belmonte at al. [204] reported about the construction and modelling of a free-standing 2W SMEP actuator. In this innovative approach, the liquid crystalline network-given reversible transformation was combined with the aforementioned layering principle. The related multilayer beam with 2W bending transformation capability was composed of a liquid crystalline EP layer, sandwiched asymmetrically between two dual-curable EP layers. The liquid crystalline EP was “programmed”, i.e., stressed, before inserting in between the two thiol-cured EP layers. These EP layers were cured by thiol-epoxy reactions at off-stoichiometry (1. curing). The actuator assembly was completed by cocoring all the layers via 1-methyimidazol catalysed homopolymerization of the EP (2. curing). The extent of bending of this multilayer beam could be controlled by the positioning, thickness and prestretching level of the liquid crystalline EP layer.

Attempt was also made to induce 2W SM effects in 3D structures using plain EPs. Wang et al. [205] argued that by exploiting the *T*_g_ difference even a 2W SMEP system can be created. The proposed system consists of a film surrounding a conical core whereby the *T*_g_ of the core (*T*_g2_) is lower than that of the film (*T*_g1_). Like the bilayer, the composite cylinder was also cocured and thus an efficient stress transfer between the covering film and core was guaranteed. Shaping occurred above *T*_g1_ and fixing under load by cooling below *T*_g2_. Upon heating the cylinder to a temperature between *T*_g2_ and *T*_g1_, its core tended to recover the original shape. The related radial contraction caused microbuckling in the cover film. So, the smooth cylinder transformed into a gear-like shape. Further temperature increases above *T*_g1_ yielded the complete recovery of the original shape. However, this concept only meets the requirement of a triple-shape system (high temperature temporary shape: smooth compressed cylinder, low temperature temporary shape: partly compressed cylinder with folded surface) and thus erroneously termed to 2W version. 

Since the basic requirement of bilayer is an asymmetric structure, this can be achieved in composites in different way (stacking sequence, reinforcement type, layering of hybrid reinforcements, etc.). This option was investigated by Basit et al. [149]. They incorporated into the composites an “active layer” consisting of CF fibres, which were used for Joule heating as trigger signal. The term “active layer” already suggests the possible use of suitable SMAs to ensure EP and related composites with 2WSM performance. In fact, this possibility was early recognized (though as matrix rubber served, [206]) and pursued by many researchers [5,152,207].

The development of the SMA-containing SMEP hybrids started with the investigations of laminated beams [178,208,209,210] followed by 2D (shell) [211,212] and 3D structures [213] and devices [178]. These works contributed to clarify open issues with respect of embedding, positioning, content, maximum straining, minimum bending radius, actuation capability-related properties, etc. of the SMA wires and foils in EP and EP-based composites. Moreover, these works were always associated with micromechanical prediction, modelling approaches [212,214,215]. Driving force of the R & D works was always the potential application of the related SMA actuators, smart adaptive materials and structures in the aerospace industry.

## 9. Modelling of SM Behaviour

Understanding and description of the behaviour of SMPs has been the subject of several papers published in the field of materials science. In this short review on the different modelling approaches we focus exclusively on the behaviours of the thermally activated SMPs especially those of the epoxy-based ones. Research on modelling of SMPs has been motivated not only by scientific interests but the design of such polymers to specific applications can also benefit from the results obtained by various modelling approaches. For example, appropriate modelling can help us to produce SMPs with higher shape fixity ratio and to tailor the kinetics of the recovery process which can, anyway, play a significant role in many areas including e.g., in biomedical and aerospace applications. Thus, there have been several attempts to describe the properties of SMPs such as those of thermoplastics and thermosets, with amorphous and/or crystalline phases, or even the properties of various composites have been also modelled. Despite the constitutional and structural versatility and diversity of the various SMP systems the description of these different SMPs is rather successful. Until recently, to describe the behaviour of the SMPs three main constitutive modelling approaches based on micro-, meso- and macro-level have been emerged and adopted. The microscale constitutive model uses a variety of details of microscale properties such as crosslinks, chain mobility and entanglements of polymer chains calculated by means of molecular dynamics (MD) or quantum chemical (QC) methods. By contrast, the macroscale constitutive models describe the material properties phenomenologically using e.g., one-dimensional (1D) or three dimensional (3D) rheological models that generally consist of a series of springs and dashpots connected in different configurations. In addition, in some cases, friction and thermal expansion elements are also involved in these models. The mesoscale modelling approaches are between the micro- and the macroscale ones: such approaches are usually detailed enough to take into account the structural heterogeneity of the materials but they are not capable of describing the materials’ properties at molecular level.

### 9.1. Macroscale Modelling Approaches

**Figure 9:**
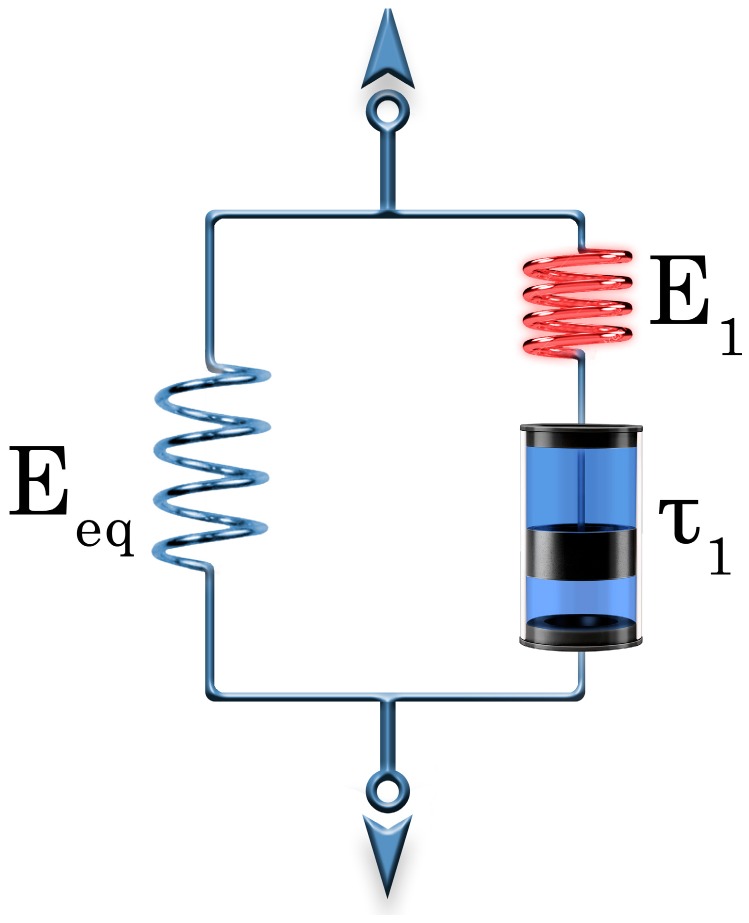
The Standard Linear Solid (SLS) viscoelastic model (also known as Zener model). *E*_eq_ and *E*_1_ is the Young’s modulus of the spring in the equilibrium and in the non-equilibrium branch, respectively, while τ represents the stress relaxation time in the non-equilibrium branch as τ = η/*E*_1_, where η is the viscosity of the liquid.

The earliest and the most generally employed macroscale constitutive model for SMPs is based on the Standard Linear Solid (SLS) model consisting of a spring and a dashpot connected in series (Maxwell element) in the non-equilibrium branch and a spring in the equilibrium branch as shown in Figure 9.

The beauty of this viscoeleastic model in addition to its simplicity is that both the shape fixity and the evolution of the recovery process in time can be qualitatively interpreted. The “equation of motion” for the SLS model is given by Equation (1).

(1) $$\sigma +\frac{\eta }{E_{1}}\frac{d\sigma }{\mathrm{dt}}=E_{\mathrm{eq}}\varepsilon +\eta {\left(1+E_{\mathrm{eq}}/E_{1}\right)}^{-1}\frac{d\varepsilon }{\mathrm{dt}}$$

where σ is the applied stress and ε is the strain.

It can be shown that according to Equation (1) the shape fixity ratio (*R*_f_) [216] and the evolution of the strain (ε) with time (t) in a free recovery process (unconstrained) under isothermal condition comes as Equations (2) and (3), respectively [217].

(2) $$R_{f}={\left(1+E_{\mathrm{eq}}/E_{1}\right)}^{-1}$$

(3) $$\ln(\varepsilon /\varepsilon_{o})=-\frac{E_{\mathrm{eq}}}{\tau E_{1}}{\left(1+E_{\mathrm{eq}}/E_{1}\right)}^{-1}t$$

where ε_o_ is the initial strain.

**Figure 10:**
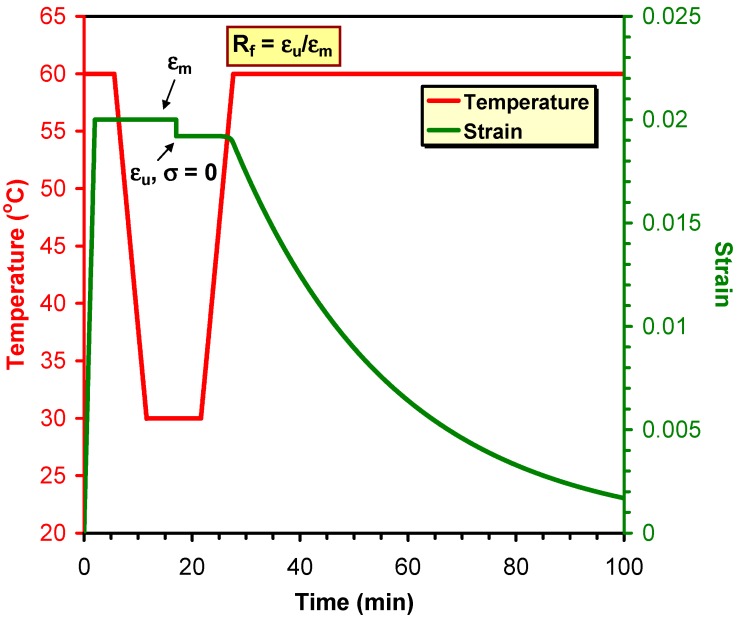
A simulation for a virtual shape memory programming based on the SLS model. Simulation parameters are: *E*_eq_ = 100 MPa, *E*_1_ = 2900 MPa, τ = 1 min, heating and cooling rate = 5 °C/min, *AF*_c_/*k*_b_ = −40,000 K where *A* is a material constant, *F*_c_ and *k*_b_ are the configurational energy and the Boltzmann’s constant, respectively.

The SM effect of SMPs is thought to be due to a drastic change in the polymer chain mobility around the glass-transition temperature (*T*_g_). Above *T*_g_, at the micro-level, the appropriate chain mobility ensures the segments of the chains to constantly rearrange themselves to reach the equilibrium state (conformation) instantaneously. However, at low temperatures (below *T*_g_) the equilibrium conformations are attained considerably more slowly. Thus, the material macroscopically “freezes” at *T* < *T*_g_, hence its viscosity is high, while it is “melted” and has low viscosity at *T* > *T*_g_. Moreover, increasing the temperature, the chain mobility is restored and shape recovery occurs. This simple macroscale constitutive approach can be applied to describe at least qualitatively the behaviour of amorphous SMPs. A simulation for a virtual shape memory programming based on the SLS model (see Figure 9), is shown in Figure 10. For the sake of simplicity only an Arrhenius-type dependence of the shifting factor on the temperature is involved in the simulation.

Various modelling reports for SMEPs employ different rheological models successfully to capture the main characteristics of shape memory behaviour. A rheological constitutive model based on the SLS and incorporation of a thermal expansion element to SLS for SMEPs was proposed by Chen et al. [218]. The time-temperature dependence of the epoxy-SMP was characterized using the Williams-Landel-Ferry equation (WLF). The model was validated and demonstrated to be capable of forecasting the measured stress-strain-temperature curves for different loading histories. Furthermore, the inspirative results also indicated that the model can characterize and predict the shape memory behaviour of SMEPs and can assist us in the design of such polymeric systems.

**Figure 11:**
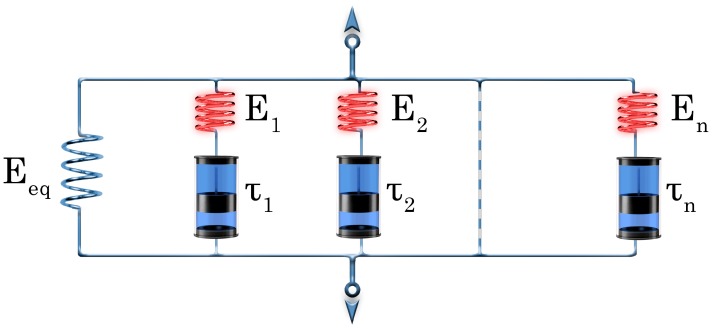
Multi-branched rheological model with the equilibrium (*E*_eq_) and non-equilibrium branches (*E*_1_, τ_1_; *E*_2_, τ_2_; … and *E*_n_, τ_n_). *E*_eq_, *E*_1_, *E*_2_, … *E*_n_ and τ_1_, τ_2_, … τ_n_ are the Young’s modulus of the springs and corresponding the stress relaxation times, respectively.

For a more realistic and quantitative description of the behaviour of the SMPs the SLS model was further extended with a series of springs and dashpot elements to render the stress relaxation time distribution observed in polymeric systems [219]. Thus, the extended model consists of an equilibrium branch and several viscoelastic non-equilibrium branches (see Figure 11).

Based on this model-frame applied for an EP-based SMP (Veriflex E), 3D constitutive relations were established and interestingly, the parameters of the 3D multi-branch model could be determined by means of standard materials tests such as stress relaxation, dynamic mechanical analysis (DMA), thermal expansion and isothermal tension tests [220]. Furthermore, finite element (FE) simulations based on the above multi-branch constitutive model revealed that the model could render the experimentally observed SM effect under different programming conditions.

A similar multi-branch rheological model was also successfully employed by Qi et al. to capture the characteristics of the amorphous acrylate- and epoxy-based SMPs [221]. In this seminal paper, the influences of the programming conditions such as the programming temperature, holding time, cooling and heating rate and shape recovery temperature were studied and interpreted in terms of the aforementioned model. The dependence of the viscoelastic properties of these SMPs on the temperature was also implemented into the model following the time-temperature superposition principle (TTSP) and using the shifting factors calculated by the Arrhenius and the WLF equations [221]. Furthermore, in order to simplify the complicated dependence of SMP behaviour on the programming and the recovery conditions, reduced programming and recovery times scaled at selected reference temperatures were also introduced [221]. In addition, the cited authors also proposed a shape memory performance map constructed by means of the multi-branch model to allow an easy prediction of the shape memory behaviour of the amorphous SMPs. The parameters of the multi-branch model were obtained experimentally from the stress relaxation experiments and DMA tests.

**Figure 12:**
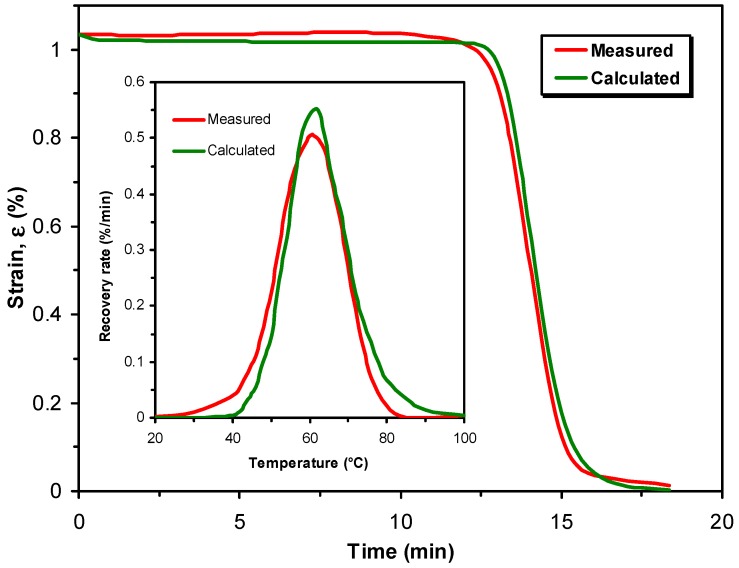
The measured and the calculated free recovery curves for a SMEP. The inset shows the measured and calculated recovery rates as a function of temperature. Reconstructed based on Ref. [225].

In the case of several SMP systems, when applying heating or cooling, the viscosity and the chain mobility of the polymer cannot follow the equilibrium state corresponding to the actual temperature, i.e., the system is out of equilibrium state, leading to structural relaxation. Thus, in addition to stress relaxation, structural relaxation has been also incorporated into the multi-branch rheological model to unravel the hysteresis in DMA traces obtained by heating and cooling process [222]. Structural relaxation was also involved in a finite deformation model proposed by Gu et al. [223]. The cited authors found that structural relaxation plays a significant role in the free recovery process of SMPs. For a more realistic description of the thermomechanical and SM effect of the SMP systems, the multi-branch rheological model can be considered with an indefinite number of Maxwell elements, i.e., with an array of relaxation times, characterized by the relaxation time spectrum. The important input parameters of the model are e.g., the shifting factors (associated with TTSP), the relaxed and unrelaxed moduli. All of these parameters can directly be determined from constant frequency DMA temperature scans. Azra et al. proposed a thermoviscoelastic constitutive model for the evaluation of the behaviour of amorphous SMPs subjected to small strains including an impressive method for the determination of the shifting factor from DMA temperature scans at constant frequency [224]. Moreover, their method was not applicable to a wide range of temperatures. Later, Kuki et al. extended the temperature range by introducing an alternative, novel approach for the determination of the parameters of the WLF and Arrhenius equations from the experimental DMA traces [225]. Subsequently, they also proposed a multi-branch viscoelastic model, which proved to be capable of rendering the kinetics of the free recovery process for epoxy- and polyurethane-based SMPs. The measured and the calculated free recovery curves for a SMEP are shown in Figure 12.

Alternatively, a fractional model derived from the SLS model has been also introduced for the prediction of isothermal and non-isothermal free recovery processes of amorphous SMPs [226]. Macroscale constitutive modelling approaches have been also successfully applied for the description of various composites including e.g., SMA-SMEP [227] and carbon nanotube (CNT)-SMEP [135] combinations.

### 9.2. Mesoscale Modelling Approaches

The first mesoscale constitutive model for the thermoset SMEPs for small strains and uniaxial loading conditions was proposed by Liu et al. [228]. According to the Liu’s model, the material is considered as a mixture of an active and a frozen phase and model calculations are based on the concept of describing the active-frozen transition as a first-order transition. Furthermore, the Liu’s model additively decomposes the strain into thermal, elastic and stored terms. The stored strain together with the volume frozen fraction is used as variables in the model to describe the change of the microstructure and to characterize the strain storage. Moreover, the cited model considers the SMPs as special elastic materials and did not take into account the time-dependent nature of the material. Later, following the work of Liu et al., Chen et al. adopted the concept of mesoscale modelling and further developed the Liu’s model for large strains [229] by introducing a multiplicative decomposition of the deformation gradients which were assumed to be dependent on the stress and temperature. Later, the linearized form of the Chen’s model for small strains was also validated for a SMEP [230]. Appropriate predictions of local stresses and strain distributions in the case of a heterogeneous structure that are important features to understand damage and failure processes, can be achieved using a mesoscale approach as it was shown by Di Prima et al. for a SMEP foam [231]. The cited authors used a hyperelastic material model in combination with finite element method to describe the compressive stress-strain response for a SMEP foam. They also demonstrated that the local tensile and local shear strain cumulative probabilities versus compressive strains obey the lognormal distributions. Such a modelling approach may assist us to predict the fatigue degradation of SMPs under cyclic loading. A mesoscale approach was also applied for the characterization of the thermomechanical behaviour of a SMA fibre-SMP composite [232].

### 9.3. Microscale Modelling Approaches

Microscale modelling of polymers and polymer networks by Quantum Chemical (QC) and especially by Molecular Dynamics (MD) methods is a rapidly expanding field and they have been applied for the characterization of a wide variety of polymeric systems. So far, the most studies have focused on the epoxies. Although these microscale modelling represent useful approaches for better understanding of the fundamental and mechanistic aspects of the SM behaviours of polymers there have been only a very limited number of reports on the EP-based SMPs.

Yang et al. studied the thermo-mechanical properties of a SMEP by means of MD simulations at different EP/curing agent (CA) ratios, i.e., at different crosslink densities [233]. According to the MD calculations performed by the cited authors, the *T*_g_ increases with the CA content and a linear correlation between the specific volume and the temperature was found both in the rubbery and in the glassy state in good agreement with the experimental results. Adopted the same concept to similar SMEP systems, Yang and co-workers [234] revealed that the free recovery time of these SMEP systems is dependent on the ratio of EP/CA and the time required for the free recovery process increases with the decreasing EP/CA ratios. Very recently, interesting SMEP networks containing reversible Diels-Alder bonds were constructed and investigated by MD method [36]. The cited authors’ theoretical studies supported the experimental fact that the flexibility of the polymer chains plays a significant role in determining the *T*_g_ and the recovery time.

In addition, multiscale modelling that combine approaches of microscale modelling with those of macroscale ones have also gained growing interest recently. For example, a statistical spring-bead based network model was proposed by Zhang et al. using MD simulation in combination with FE method to capture the properties of EP-based smart polymeric systems [235].

## 10. Outlook and Future Trends

Though chemistry of EP curing is well known (so, we have all the necessary tools to tailor *T*_g_ and thus *T*_trans_ upon request), even here we can trace some less explored possibilities, such as dynamic bonding via vitrimer and click chemistry, semi- and full-IPNs and co-networks. Similar to EPs with dynamic bonds, EP-based semi-IPNs may combine SM and self-healing functionalities. Components of SMEPs may be derived from selected renewable resources.

Vivid development can be expected for SMEP composites. The target with traditional reinforcements is to find their necessary amount and lay-up which yields the highest storage of elastic energy thereby maintaining high deformability for a given loading mode. Benefits of reinforcement hybridizations will also be checked with respect to SM and especially to 2W SM performance. To support the related research, SM cycling tests will be followed by suitable non-destructive testing techniques. Mapping of the failure mode will help to deduce guide lines for packaging/deploying, i.e., temporary shaping/recovery.

Interfacial engineering of composites with respect to SM and other functionalities, such as self-healing, is yet hardly explored though seems to be very promising [236,237].

Research work will be intensified to replace direct heating by other means for the shaping and recovery. For this purpose, the inherent properties of traditional reinforcements, such as the electric conductivity of CF, will be combined with other desired properties (e.g., heat conductivity) given by (nano)fillers. Incorporation of nanofillers, such as CNF, into suitable EP resin formulation would allow us the use of the emerging 3D printing technology (i.e., inkjet printing) to manufacture SMEP composites [238]. Nowadays, when the 3D printed (multimaterial) architectures have additional functionality, such as SM, they are often referred to 4D systems.

The combination of SMEP with SMA remains a hot topic with many challenges (optimum amount of SMA including its structuring, adhesion of SMA to the matrix, modelling the properties). Simulation and modelling studies using different approaches will be further the drivers of the SMEP-based material developments in the future.

The future belongs to “active” and multifunctional SMEPs and related composites [96,239]. “Active” means the adaptability of the material to give an adequate response to varying external stimuli, whereas multifunctionality involves a combination of non-interrelated properties.

SMEP composites are promising candidate materials for various parts of space- (e.g., solar arrays, antennas, booms, deployable panels, reflectors) [152,240,241] and aircrafts (different morphing concepts for wing parts and skins) [242,243]. This is owing to their good SM properties, excellent stiffness, strength and environmental durability.
